# Exo-miR-1290-induced by COX-2 overexpression promotes cancer-associated fibroblasts activation and tumor progression by CUL3-Nrf2 pathway in lung adenocarcinoma

**DOI:** 10.1186/s12964-023-01268-0

**Published:** 2023-09-18

**Authors:** Xiaoming Bai, Jiaofang Shao, Tinghong Duan, Xue Liu, Min Wang, Xuanya Li, Qiang You, Zhiyuan Zhang, Jinshun Pan

**Affiliations:** 1 Department of Pathology, Nanjing Medical University, 101Longmian Avenue, Jiangning District, Nanjing, 211166 P.R. China; 2 Department of Bioinformatics, Nanjing Medical University, 101Longmian Avenue, Jiangning District, Nanjing, 211166 P.R. China; 3 Department of Biotherapy, the Second Affiliated Hospital of Nanjing Medical University, Nanjing, 210011 P.R. China

**Keywords:** COX-2, Exo-miR-1290, CUL3, Nrf2, CAFs activation, LUAD

## Abstract

**Background:**

Cancer-associated fibroblasts (CAFs) are critically involved in tumor progression by maintaining extracellular mesenchyma (ECM) production and improving tumor development. Cyclooxygenase-2 (COX-2) has been proved to promote ECM formation and tumor progression. However, the mechanisms of COX-2 mediated CAFs activation have not yet been elucidated. Therefore, we conducted this study to identify the effects and mechanisms of COX-2 underlying CAFs activation by tumor-derived exosomal miRNAs in lung adenocarcinoma (LUAD) progression.

**Methods:**

As measures of CAFs activation, the expressions of fibroblasts activated protein-1 (FAP-1) and α-smooth muscle actin (α-SMA), the main CAFs markers, were detected by Western blotting and Immunohistochemistry. And the expression of Fibronectin (FN1) was used to analyze ECM production by CAFs. The exosomes were extracted by ultracentrifugation and exo-miRNAs were detected by qRT-PCR. Herein, we further elucidated the implicated mechanisms using online prediction software, luciferase reporter assays, co-immunoprecipitation, and experimental animal models.

**Results:**

In vivo, a positive correlation was observed between the COX-2 expression levels in parenchyma and α-SMA/FN1 expression levels in mesenchyma in LUAD. However, PGE2, one of major product of COX-2, did not affect CAFs activation directly. COX-2 overexpression increased exo-miR-1290 expression, which promoted CAFs activation. Furthermore, Cullin3 (CUL3), a potential target of miR-1290, was found to suppress COX-2/exo-miR-1290-mediated CAFs activation and ECM production, consequently impeding tumor progression. CUL3 is identified to induce the Nuclear Factor Erythroid 2–Related Factor 2 (NFE2L2, Nrf2) ubiquitination and degradation, while exo-miR-1290 can prevent Nrf2 ubiquitination and increase its protein stability by targeting CUL3. Additionally, we identified that Nrf2 is direcctly bound with promoters of FAP-1 and FN1, which enhanced CAFs activation by promoting FAP-1 and FN1 transcription.

**Conclusions:**

Our data identify a new CAFs activation mechanism by exosomes derived from cancer cells that overexpress COX-2. Specifically, COX-2/exo-miR-1290/CUL3 is suggested as a novel signaling pathway for mediating CAFs activation and tumor progression in LUAD. Consequently, this finding suggests a novel strategy for cancer treatment that may tackle tumor progression in the future.

Video Abstract

**Supplementary Information:**

The online version contains supplementary material available at 10.1186/s12964-023-01268-0.

## Background

Lung cancer is the second most commonly diagnosed cancer and the leading cause of cancer death, with an estimated 1.8 million deaths (18%) worldwide [1]. LUAD is one of most common types of lung cancer, with a global 5-year survival rate ranging from 10–20% worldwide. Despite advancements in medical research, this survival rate has remained relatively unchanged over the past three decades [1, 2]. Genetic studies reveal significant correlationship between the tumor microenvironments (TME) and tumor progression in lung cancer patients [3–5]. Therefore, it is necessary to identify new mechanisms that affect TME to improve therapy and patient survival in lung cancer.

Cancer-associated fibroblasts (CAFs) are the most abundant stromal components in TME [6, 7]. Numerous studies have identified that CAFs are prominent in shaping the extracellular matrix (ECM), particularly the α-SMA expression, increasing the synthesis of ECM, and improving the remodeling of ECM, and cancer pathogenesis [6–9]. However, the mechanism underlying CAFs activation is still to be clarified in LUAD. Cyclooxygenase-2 (COX-2), and its downstream products are well known as important factors associated with carcinogenesis and progression in many types of cancers, and COX-2 inhibition also sensitizes cancer cells to radio- and chemotherapy [10, 11]. Numerous studies have revealed that COX-2 was positively associated with ECM synthesis in various cancers [12–14]. However, the role and mechanisms of COX-2 on CAFs activation are still unclear.

Our previous studies has shown that COX-2 upregulates cell growth and invasion in LUAD [15]. Nevertheless, PGE2, the major product of COX-2, suppresses all hallmarks of pro-fibrotic fibroblast activity, including survival, migration and ECM restructure ability [16]. There seem to be some other crosstalks between cancer cells and CAFs induced by COX-2, except PGE2.

Exosomes are small (30–150 nm) vesicles containing sophisticated RNA and protein cargo, secreted by all cells in vitro and in vivo [17]. Numerous studies have pointed out that exosomes mediate the TME regulation to promote cancer development and progression [18–20]. MicroRNAs (miRNAs) are small, non coding RNAs, that inhibit target genes translation [21]. Recently, exosomal miRNAs have been shown to contribute to immunosuppression, chemoresistance, and metastasis in many cancers [17, 18, 20, 21]. However, the roles and mechanisms of some exo-miRNAs on COX-2-mediated tumor progression are still unclear.

This study aimed to examine the role and mechanisms of COX-2-mediated CAFs activation in LUAD, to investigate whether exosomes contribute to COX-2-mediated CAFs activation and ECM production, and to verify what types of exo-miRNAs are involved in the process. The results of our study provide potential therapeutic approaches for the treatment of LUAD by focusing on the modulation of the TME in lungs.

## Methods

### Patients and specimens

Primary surgical specimens were from 30 patients (aged from 52 to 83; average, 64) who were diagnosed clinically for LUAD, from Nanjing Chest Hospital in 2014. None of participants developed distant metastasis. All of them were approached for participation in the project. All experimental protocols were approved by the Human Ethics Committee of Nanjing Medical University, including any relevant details. The work conforms to the provisions of the Declaration of Helsinki in 1975. Written informed consent was obtained from all the donors for use of these samples in research. Resected specimens were fixed with 10% neutral-buffered formalin and embedded in paraffin blocks. The diagnosis and histological grade of all the cases were confirmed independently by two pathologists, based on World Health Organization (WHO) classification.

### Cell culture and reagents

Human LUAD cell lines (A549), mouse embryonic fibroblast cell lines (NIH-3T3) and human embryonic kidney 293 T (HEK-293 T) were obtained from the American Type Culture Collection (ATCC, Manassas, VA, USA), and cultured in Dulbecco’s modified Eagle’s medium (DMEM) supplemented with 10% fetal bovine serum, penicillin (100 U/mL), and streptomycin (100 ng/mL). Human normal lung fibroblast cell line (MRC-5) was obtained from KeyGEN BioTECH (Nanjing, China), cultured in minimum essential medium (MEM) with Nonessential amino acids and pyruvic acid, and supplemented with 10% fetal bovine serum, penicillin (100 U/mL), and streptomycin (100 ng/mL). All cell lines were maintained in a 37 °C incubator with 5% CO2. All mediums, antibiotics and fetal bovine serum were obtained from Invitrogen (Carlsbad, CA, USA).

### Animals

All animals (female nude mice) were treated in accordance with the guidelines of the Institutional Animal Care and Use Committee at Nanjing Medical University. The animals were fed food and water ad libitum, and housed at 22 °C with a 12-h light–dark cycle. The typical diet was acquired from Xietong Biotechnology Co. Ltd (Jiangsu, China). All animal experiments were conducted following the National Institutes of Health Guidelines for the care and use of Laboratory animals (NIH Publications No. 8023, revised 1978). All animal studies complied with the ARRIVE guidelines.

### Plasmid, miRNA mimics and siRNA transfections

The pcDNA3-based plasmid encoding human NFE2L2 (Nrf2) was obtained from the nonprofit plasmid repository, Addgene (Cambridge, MA USA). The miRNA mimics were obtained from Ribobio Biotechnology Co., Ltd (Guangzhou, China). SiRNAs were acquired from Santa Cruz Biotechnology (Santa Cruz, CA, USA): Nrf2 siRNA (h) sc-37030, Nrf2 siRNA (m) sc-37049, CUL-3 siRNA (h) sc-35130, CUL-3 siRNA (m) sc-35131.

The cells (3 × 10^5^) were seeded and grown in 6-well culture plates for 24 h before transfection with the above plasmids, miRNA mimics, or siRNAs using Lipofectamine 3000 (Invitrogen, Carlsbad, CA, USA).

### Lentiviral infection

The sequences encoding COX-2 was cloned from COX-2-pcDNA plasmid. Human CUL3 cDNA ORF Cloning Vector was obtained from SinoBiological Inc (Beijing, China). Both cDNAs were cloned into the pLJM1 lentivirus vectors (Addgene, Palo Alto, CA, USA). To produce the virus, the plasmids were co-transfected into HEK-293 T cells, to produce the virus according to the manufacturer’s instructions. To obtain a stable COX-2- or CUL3-overexpressing cell line, lentivirus-containing supernatant was harvested and used to infect A549 or NIH-3T3 cells. COX-2-overexpressed A549 cells (A549-COX-2) were developed based on A549 cells, whereas CUL3-overexpressed NIH-3T3 cells (3T3-CUL3) were developed based on NIH-3T3 cells.

### Exosome extraction

A549 cells were cultured in the complete medium until they reached 60% confluence. Subsequently, the medium was replaced with DMEM medium supplemented with 10% FBS (exosomes deletion by ultracentrifugation). After an additional 48 h of incubation, the conditioned medium of A549 was collected and centrifuged at 300 g for 10 min and 2,600 g for 10 min at 4℃ to remove dead cells and cellular debris. Thereafter, the supernatant was centrifuged at 10,000 g for 1 h and 100,000 g for 3 h. The pellet was resuspended with PBS and centrifuged at 100,000 g for 2 h, then resuspended again with PBS and stored at -80℃.

The mice were anaesthetized and 500 µL of blood was taken from the heart of each animal. After centrifugation, the upper plasma was collected from each group, and the exosomes from plasma were extracted by Total exosome isolation kits (#4484450) from Thermo Fisher Scientific Inc (Carlsbad, California, USA).

### Exosome identification and tracing

The exosomes were observed by negative staining by JEM-1400 Flash transmission electron microscope (JEOL Ltd., Japan). Consequently, the exosomes were quantified by BCA assays and identified by Western blotting analysis.

For exosome-tracing experiments, exosomes in CM was obtained as described above. After labeled with DiO (Beyotime, China), the exosomes were added to NIH-3T3 cells for 30 min, and then the recipient cells were observed by Lsm710 confocal laser scanning microscopy (CarlZeiss, Germany).

### RNA isolation and quantitative real-time PCR analysis

Total RNA was isolated using the Trizol reagent, according to the manufacturer’s instructions. The exosomal RNA was isolated through the Trizol reagent, supplemented with glycogen.

Typically, 0.5 μg of total RNA was reverse transcribed For each sample using the HiScript II Reverse Transcriptase Kits (Nanjing Vazyme Biotech Co., Ltd). Consequently, real-time PCR analysis was performed using the Power SYBR Green PCR master mix (Roche Diagnostics, #04913914001, Indianapolis, IN, USA). All the miRNAs RT and PCR primers were obtained from Ribobio Biotechnology Co. Ltd (Guangzhou, China). The primers of KEAP1, Nrf2, CUL3, and β-actin were obtained from Tsingke Biotechnology Co., Ltd (Beijing, China). The sequences were as shown in Table 1.

**Table 1:** The DNA sequences of Primers

Gene	Species	Sequences
KEAP1	Human & Mouse	Forward Sequence CAACTTCGCTGAGCAGATTGGC Reverse Sequence TGATGAGGGTCACCAGTTGGCA
CUL3	Human	Forward Sequence TCGACAGCTCACACTCCAGCAT Reverse Sequence GTGCTTCCGTGTATTAGAGCCAG
CUL3	Mouse	Forward Sequence GAGTTCAGGCAACATCTACAGGC Reverse Sequence GCACTTTGGTGTGGCTGACTGA
Nrf2	Human	Forward Sequence CACATCCAGTCAGAAACCAGTGG Reverse Sequence GGAATGTCTGCGCCAAAAGCTG
Nrf2	Mouse	Forward Sequence CAGCATAGAGCAGGACATGGAG Reverse Sequence GAACAGCGGTAGTATCAGCCAG
β-actin	Human	Forward Sequence CACCATTGGCAATGAGCGGTTC Reverse Sequence AGGTCTTTGCGGATGTCCACGT
β-actin	Mouse	Forward Sequence CATTGCTGACAGGATGCAGAAGG Reverse Sequence TGCTGGAAGGTGGACAGTGAGG

All treatments and conditions were performed in triplicate to calculate statistical significance

### Western blotting

Cells were treated with pharmacological agents at various times, and subsequently collected into lysis buffer. MG132 and NS-398 were obtained from MedChemExpress (Shanghai, China). Equal amounts of total proteins (20 μg) were separated by SDS-PAGE, transferred onto nitrocellulose membranes, and immunoblotted with appropriate antibodies. The immunoreactivity was detected by ECL and analyzed using Image J software. The following antibodies were commercially obtained: the anti-Nrf2 (#sc-365949) was obtained from Santa Cruz Biotechnology; the Anti-CUL3 (#10450) was obtained from Cell Signaling Technology (Danvers, MA, USA); the anti-α-SMA (#ab7817) was obtained from Abcam plc (Cambridge, CB2 0AX, UK). Anti-COX-2 (#160112) was from Cayman Chemical Co (Ann Arbor, MI, USA). Anti-KEAP1 (#A00514-3) and anti-FN1 (#BA1771) antibodies were obtained from Boster Biological Technology co. (Wuhan, China); anti-FAP-1 antibody (#SAB4500839) was obtained from Sigma Chemical Co. (St. Louis, MO, USA); the anti-β-actin antibody was obtained from Bioworld Technology (Atlanta, Georgia, 305, USA). Anti-CD9 (20597–1-AP), CD81 (66866–1-Ig), CD63 (67605–1-Ig), TGS101 (28283–1-AP) were obtained from Proteintech Group, Inc (Wuhan, China).

### mRNA sequence (mRNA‑seq)

NIH-3T3 cells were treated with exosomes from A549-Con or A549-COX-2 48 h before total RNA was extracted with TRIzol (Life Technologies, USA). After quality control with Nanodrop 2000, total RNA was temporarily stored at -80 °C, and high-throughput mRNA sequencing was performe using Illumina HiSeq 2500 system from Tsingke Biotechnology Co., Ltd (Beijing, China).

Differential expression analysis of two groups was performed using the DESeq2 R package (1.26.0). DESeq2 provide statistical routines for determining differential expression in digital gene expression data using a model based on the negative binomial distribution. The resulting *P* values were adjusted using Benjamini and Hochberg’s approach for controlling the false discovery rate. The genes with FDR < 0.05 & |log2(foldchange)|≥ 1 found by DESeq2 were assigned as differentially expressed. Gene Ontology (GO) enrichment analysis of differentially expressed genes (DEGs) was implemented by Goseq R packages based on Wallenius non-central hyper-geometric distribution.

### Co-immunoprecipitation

Cells were lysed in NETN buffer (20 mM Tris, at pH 8.0, 100 mM NaCl, 1 mM EDTA and 0.5% NP-40). For all samples, 1000 μg lysate was incubated with Protein G Sepharose beads and then followed by the indicated antibodies according the manufacturer’s instruction. Immunoprecipitates were resolved by immunoblotting with the indicated antibodies: Anti-Nrf2 (#ab62352) was obtained from Abcam plc (Cambridge, CB2 0AX, UK), where as Anti-HA (#H3663) was acquired from Sigma Chemical Co. (St. Louis, MO, USA).

### Tumor xenograft models

Four-week-old female nude mice were injected into the flanks with 5 × 10^6^/0.1 mL of various A549 and NIH-3T3 cell lines (1:1). The bi-dimensional tumor measurements were recorded every two days. Subsequently, tumor volume (mm^3^) was calculated using the following formula: V = 1/2L × W^2^ (L: length, W: width).

In some experiments, the mice were treated with intratumor injection of exosomes (4 injections of 100 μg exosome protein in PBS every 48 h) [17]. Mice were euthanized, tumor xenografts were removed, and the specimens were fixed with neutral-buffered 10% formalin and embedded in paraffin blocks.

### Immunohistochemical staining

Sections (4 µm) of tumor blocks were used for immunohistochemical analysis. The sections were treated with primary antibodies. The slides were stained with diaminobenzedine solution (DAB), and then counterstained with haematoxylin. The slices were photographed with a Nikon Microscope and Image Pro Plus analysis system. Four high-power views (400 ×) were selected randomly from each sample in a blinded manner; the level of integrated OD (IOD) was estimated and presented as mean ± SEM.

### Dual-luciferase reporter assay

The dual-luciferase reporter assay system was obtained from Promega Corporation (Madison, WI, USA). The 3’-UTR-luciferase reporter constructs containing the 3’-UTR regions of CUL3 with the wild-type and mutant binding sites of miR-1290 were cloned into pMIR-GLO vectors. Furthermore, the promoter regions of FAP-1 and FN1 with the binding sites of Nrf2 (anti-oxidant response element, ARE), were synthesized by Tsingke Biotechnology Co., Ltd (Beijing, China). Luciferase activities were measured 24 h after transfection using the Dual Luciferase Reporter Assay System. All experiments were performed with three independent replicates showing consistent results each time.

### Chromatin immunoprecipitation assay

According to the manufacturer’s instructions, Chromatin immunoprecipitation (ChIP) assays were performed using a Thermo Scientific Pierce Agarose ChIP Kit (#26156, Carlsbad, California, USA). The chromatin was precipitated with nonspecific IgG antibodies or ChIP-grade rabbit Anti-Nrf2 (Abcam). Subsequently, DNA was extracted, and PCR was performed using primers for FAP-1 and FN1 promoter fragments containing ARE domain. Primers of FAP-1 and FN1 promoters were as followed:

- FAP-1: the forward primer: 5’-AAAGAATGTAGCCCCTGCCT-3’; the reverse primer: 5’-AACATGCCACTTCCCTCAGT-3’;
- FN1: the forward primer: 5’-CCTAAGCTTTGCCTCTCTCACTT-3’; the reverse primer: 5’- GTCACAAAACCAGACTCGTTGA-3’

### Statistical analysis

Spearman’s correlation analysis approach was applied to the investigation of the co-expression between COX-2 and the genes that were involved in the intersection. Moreover, the overall survival (OS) of miR-1290 was analyzed using the Kaplan–Meier plotter in the TCGA database.

Other data are presented as mean ± SEM. *P*-values were calculated by one-way ANOVA or Student’s t-test for unpaired samples using the GraphPad Prism software. The results were considered significant at *P* < 0.05.

## Results

### COX-2 promoted CAFs activation in LUAD

**Fig. 1:**
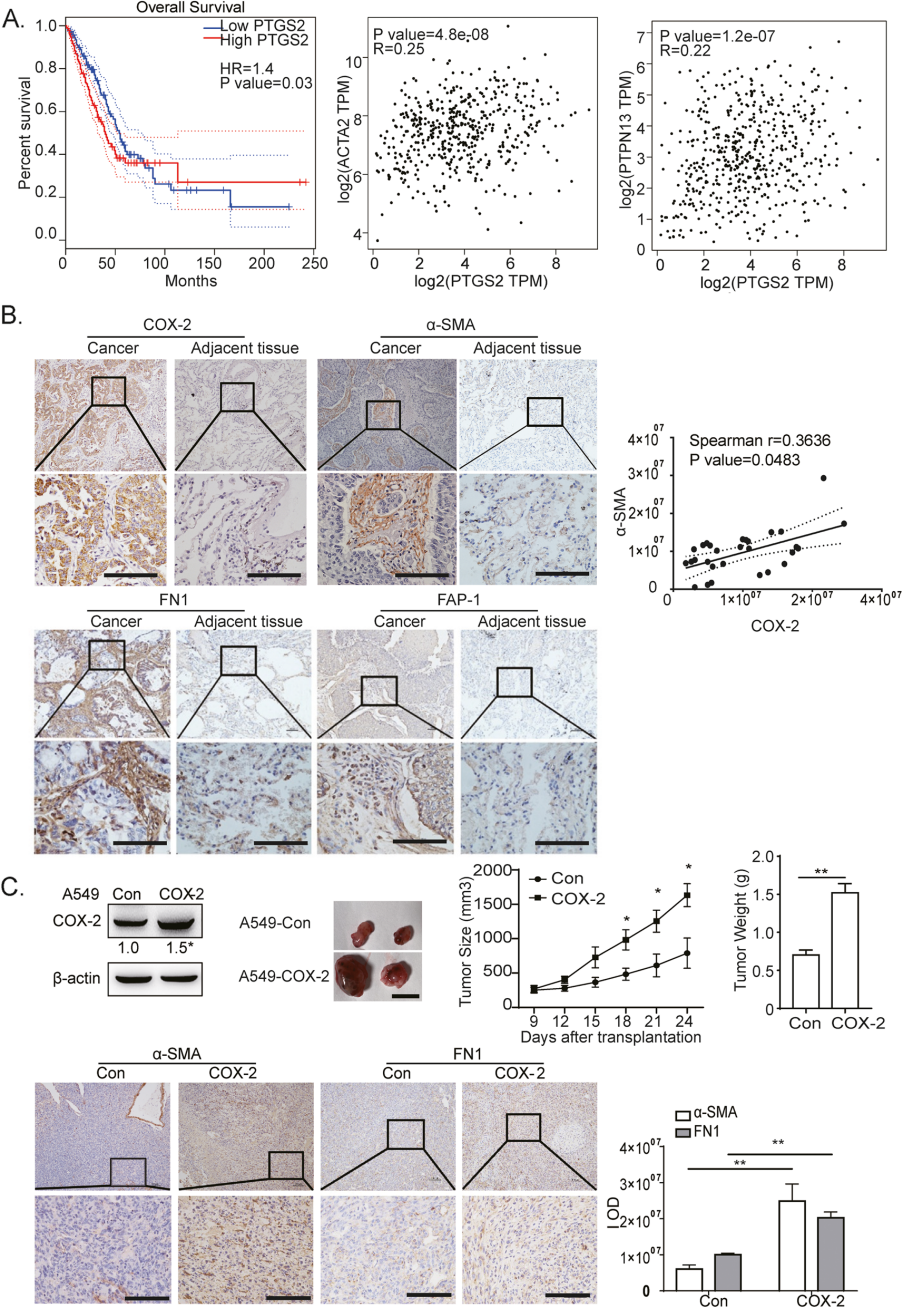
COX-2 upregulated CAFs activation in LUAD. **A** The association of COX-2 (Also known as prostaglandin-endoperoxide synthase 2, PTGS2) with overall survival in LUAD in TCGA database using online Kaplan Meier Plotter tool. The correlations between COX-2 and α-SMA (ACTA2) or FAP-1 (PTPN13) in LUAD in TCGA database. **B** Representative immunohistochemical images of LUAD tissues stained with anti-COX-2, anti-α-SMA, anti-FN1, and anti-FAP-1 antibodies (Low power view 10 × , High power view 40 × , Scale bars = 100 μm). The IOD level was determined using Image Pro Plus software. Spearman’s correlation analysis of COX-2 and α-SMA expression was shown. **C** Representative immunohistochemical images of COX-2 overexpressed lung cancer xenografts stained with anti-α-SMA and anti-FN1 antibodies (Low power view 10 × , High power view 40 × , Scale bars = 100 μm). Overexpression of COX-2 in A549 cells was identified with Western blotting. Cells with or without COX-2 overexpression were injected subcutaneously. Representative pictures of tumors, tumor size, and weight were obtained at the indicated time points (*n* = 4, Bar = 10 mm). The IOD levels of α-SMA and FN1 were determined using Image Pro Plus software. Data were presented as the means ± SEM. **P < 0.01 compared to the Con group

In our previous studies, it was clarified COX-2 is highly expressed in about 80% of LUAD cases. Additionally, our finding demonstrated that COX-2 promoted cell proliferation, migration and invasion in LUAD cells [15]. Currently, COX-2 expression was analyzed in LUAD and Lung squamous cell carcinoma (LUSC) in the TCGA database. Higher levels of COX-2 expression were found to be associated with a poor prognosis in LUAD; however, this association was not detected in LUSC. To observe whether COX-2 promotes CAFs activation in mesenchyma, we went on to detect the expressions of activated fibroblast markers: α-SMA and FAP-1, and the expression of FN1 in mesenchyma in LUAD tissues. Our founding indicated that COX-2 was positively associated with both α-SMA and FAP-1 in LUAD, but not LUSC, in TCGA database (Fig. 1A, Additional file 1). Immunohistochemical assays showed that the expressions of α-SMA, FN1, and FAP-1 were particularly higher in mesenchyma of LUAD tissues, than in adjacent tissue. Furthermore, there was a positive correlation between COX-2 expression and α-SMA in mesenchyma (Fig. 1B).

To detect whether COX-2 promotes CAFs activation, A549 cells were stably overexpressed with COX-2, and then subcutaneously injected to prepare lung cancer xenografts. Figure 1C shows that COX-2 overexpression in cancer cells significantly increased α-SMA and FN1 expression in the mesenchyma of implanted tumor tissues. These data indicate that COX-2 can promote CAFs activation and FN1 expression in LUAD.

### Tumor-derived exosomes contributed to COX-2-mediated CAFs activation in LUAD

**Fig. 2:**
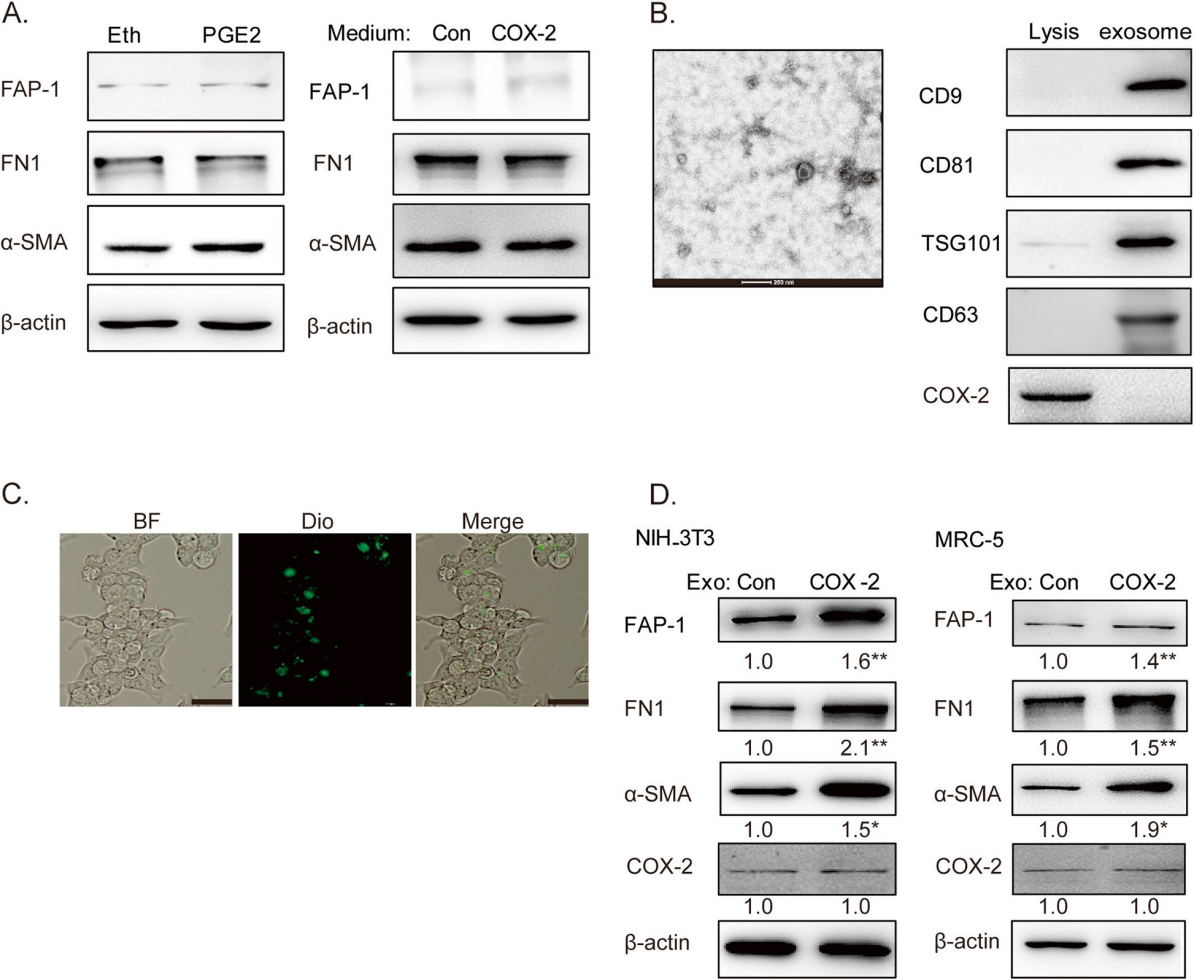
Tumor-derived exosomes contributed to COX-2-mediated CAFs activation in LUAD. **A** Western blotting assays of NIH-3T3 cells detected with anti-α-SMA, FN1, and FAP-1 antibodies after treatments with condition medium of A549-Con or A549-COX-2 cells, or PGE2 5 μM for 48 h. **B** Exosome identification. Exosomes were identified by transmission electron microscope and the expressions of exosome markers (CD9, CD81, TGS101, CD63) and COX-2 were then assessed by Western blotting. **C** Exosome tracing. Exosomes were labeled with Dio, and the recipient cells were observed by confocal laser scanning microscopy. Scale bars = 100 μm (40 ×). **D** Western blotting assays of fibroblast cells detected with anti-α-SMA, FN, FAP-1 and COX-2 antibodies after treatments with A549-Con or A549-COX-2 exosomes. β-actin was used as an internal reference. Data were presented as the means ± SEM from three independent experiments. *P < 0.05, **P < 0.01 compared to A549-Con group

CAFs are activated by tumor cells secreting cytokines, chemokines, or some other steps [20, 22–24]. To detect the mechanism of COX-2-mediated CAFs activation, PGE2, the main product of COX-2, was treated on NIH-3T3 cells. However, PGE2 did not increase the expressions of α-SMA, FN1, and FAP-1. Furthermore, treatment with the condition medium of A549-COX-2 has little effect on the expressions of fibroblast markers in NIH-3T3 in 48 h (Fig. 2A). These data suggest that some other ways, expect cytokines or PGE2, may play a major role in COX-2-mediated CAFs activation in LUAD.

As it is well known that exosomes mediate a lot of interaction between cancer cells and stroma [21], we tried to detect that whether exosomes play essential roles in COX-2-mediated CAFs activation in LUAD. As was shown in Fig. 2B, C, exosomes were isolated from the medium from A549-Con or A549-COX-2 by ultracentrifugation, and Dio-labeled exosomes were observed within NIH-3T3 cells. The exosomes derived from A549-COX-2 cells upregulated the expressions of α-SMA, FN1, and FAP-1 in both MRC-5 and NIH-3T3 cells (Fig. 2D), suggesting that tumor-derived exosomes contribute to COX-2-mediated CAFs activation in LUAD. Additionally, exosome treatment had no effects on COX-2 expression in the fibroblasts, suggesting that COX-2 played its role in the parenchyma, and not mesenchyma.

### Exo-miR-1290 was responsible for COX-2-mediated CAFs activation in LUAD

**Fig. 3:**
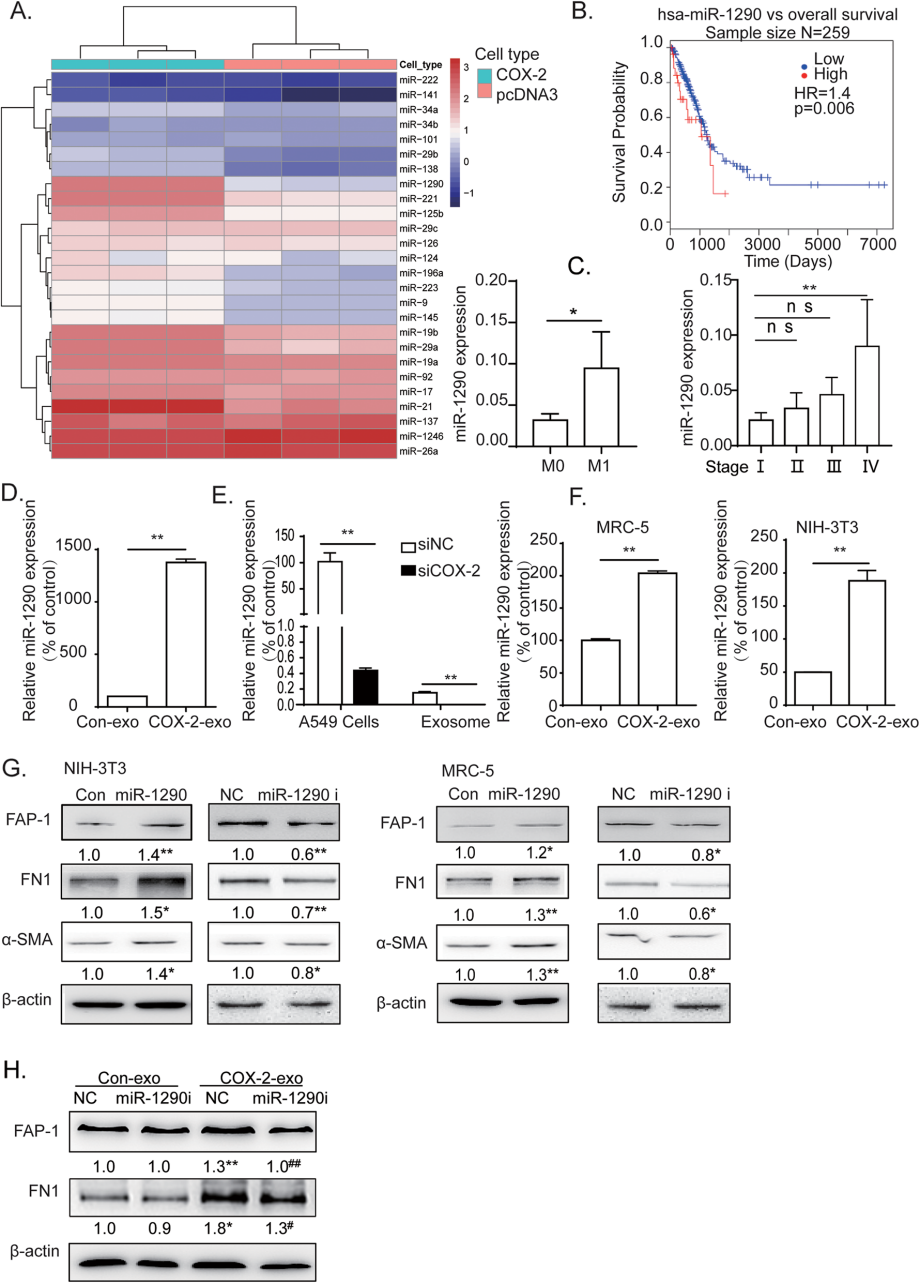
Exo-miR-1290 is responsible for COX2-mediated CAFs activation. **A** The Heatmap representation of quantification of 26 miRNAs profile of A549-con or A549-COX-2 cells. **B** The association of miR-1290 expression with overall survival in LUAD in TCGA database using online Kaplan Meier Plotter tool. **C** The expression levels of miR-1290 were analyzed by the TCGA database, according to metastatic (M1) or non-metastatic (M0), or different stages of LUAD. **D** qRT-PCR assays of miR-1290 expression in A549-COX-2 exosomes, compared with A549-Con exosomes. **E** qRT-PCR assays of miR-1290 expression in siCOX-2-transfected A549 and its exosomes, compared with siRNA negative control. **F** qRT-PCR assays of miR-1290 expression in fibroblasts treated with A549-COX-2 exosomes, compared with those treated with A549-Con exosomes. U6 was used as internal reference. Data are presented as the mean ± SEM from three independent experiments, **P < 0.01, compared with the corresponding control. **G** Western blotting assays of fibroblasts detected with anti-α-SMA, FN1, and FAP-1 antibodies after treatments with miR-1290 mimic or inhibitor, compared with corresponding control. **H** Western blotting assays of NIH-3T3 cells were detected with anti-FN1 and FAP-1 antibodies after treatments with miR-1290 inhibitor, exosomes, or both. β-actin was used as internal reference. Data were presented as the means ± SEM from three independent experiments. *P < 0.05, **P < 0.01 compared with corresponding control; #*P* < 0.05, ##*P* < 0.01 compared with COX-2-exo groups

It is well known that extracellular vesicles contain different forms of RNA and DNA. As miRNAs contain more than 70% of RNA species in exosomes [21], we therefore observe whether some exo-miRNAs play major roles in COX-2-mediated CAFs activation. The expressions of 26 miRNAs, which were the most commonly involved miRNAs in tumor progression in LUAD [20, 21, 25, 26], were detected in A549-Con or A549-COX-2. Among them, miR-1290 was the most upregulated miRNA, reaching a 100-fold increment after COX-2 overexpression (Fig. 3A).

To observe the role of miR-1290 in CAFs activation, the miR-1290 expression was analyzed in lung cancer tissues in TCGA database and various lung cancer cells. On the one hand, higher expression of miR-1290 was associated with a poor prognosis in TCGA database (Fig. 3B); miR-1290 expression increased significantly in metastatic period, and the end stage in LUAD, but not in LUSC (Fig. 3C, Additional file 2). On the other hand, miR-1290 expression was detected in exosomes isolated from A549-Con or A549-COX-2. Figure 3D shows that COX-2 overexpression increased exo-miR-1290 expression obviously. Reversely, COX-2 inhibition decreased miR-1290 expression in LUAD cells. In addition, siCOX-2, and COX-2 inhibitor NS-398, completely abrogated miR-1290 expresion in exosomes (Fig. 3E, Additional file 2). These data suggest that exo-miR-1290 may be involved in COX-2-mediated LUAD progression.

We then identify the effects of exo-miR-1290 on CAFs activation. Firstly, the miR-1290 expression was detected in MRC-5 and NIH-3T3 cells after exosomes treatment. The exosome derived from A549-COX-2 significantly increased miR-1290 expression in both fibroblasts (Fig. 3F). Secondly, miR-1290 mimic triggers CAFs activation and FN1 production in both fibroblast cells, while miR-1290 inhibitor decreased the expressions of CAF activation markers (Fig. 3G). Thirdly, miR-1290 inhibitor reversed the CAFs activation and FN1 production mediated by exosomes from A549-COX-2, although miR-1290 inhibitor had little effect on FAP-1 and FN1 expression regulated by A549-Con exosomes, which may be related to the low basal level of miR-1290 in fibroblasts (Fig. 3H). These data reveal that exo-miR-1290 may play a significant role in COX-2-mediated CAFs activation in LUAD.

### Exo-miR-1290 promoted CAFs activation by upregulating Nrf2 expression in fibroblasts

**Fig. 4:**
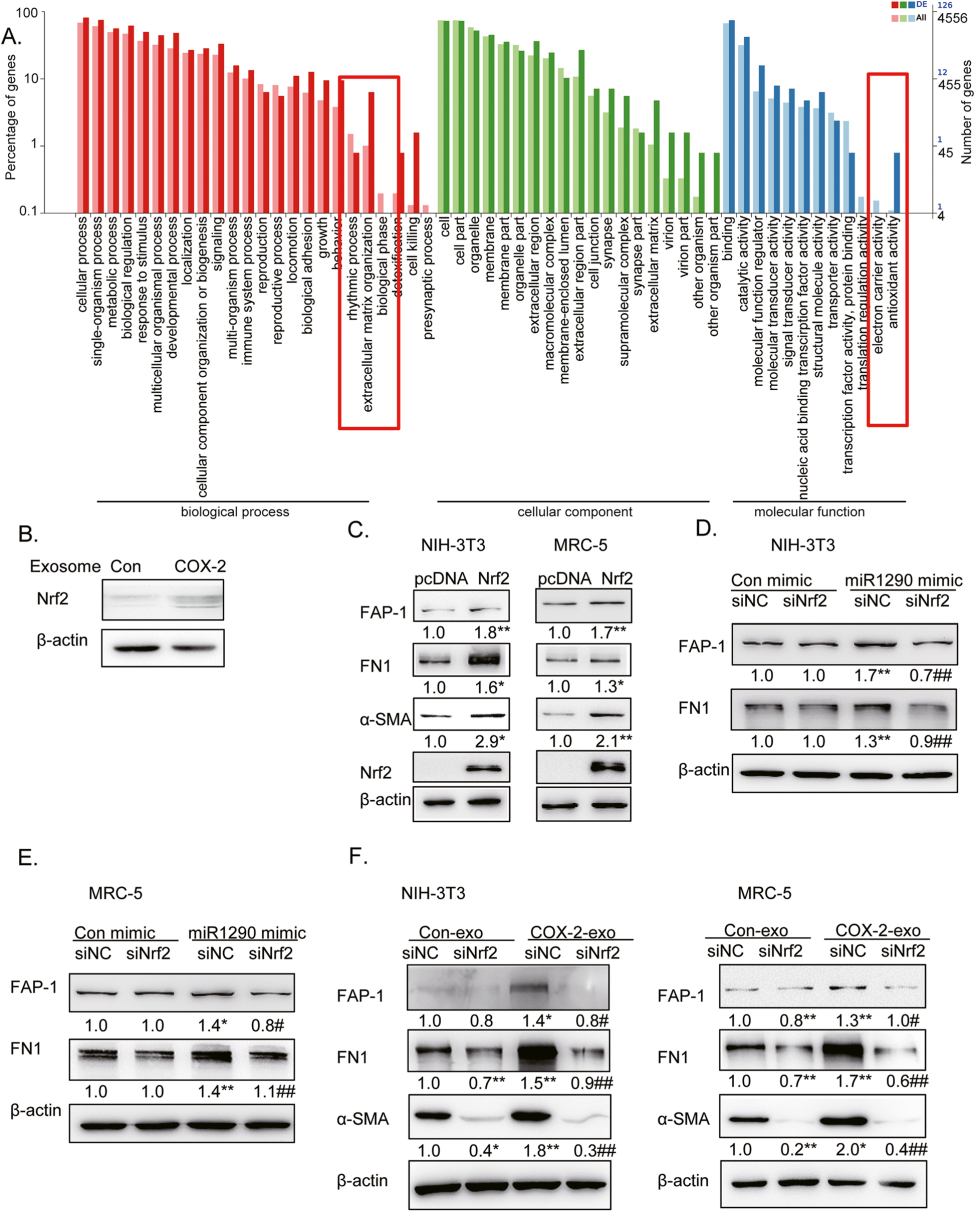
Exo-miR-1290 promoted CAFs activation by upregulating Nrf2 expression in fibroblasts. **A** GO enrichment analysis showed that the antioxidant activities were involved in the COX-2 exosomes-mediated CAFs activation. **B** Western blotting assays of NIH-3T3 cells detected with anti-Nrf2 antibody after treatments with exosomes from A549-Con or A549-COX-2 cells. **C** Western blotting assays of fibroblasts detected with anti-α-SMA, FN1, and FAP-1 antibodies after treatments with Nrf2 overexpression. **D**, **E** Western blotting assays of fibroblasts detected with anti-FN1, and FAP-1 antibodies after treatments with siNrf2 and/or miR-1290 mimic transfection. (D. NIH-3T3, E. MRC-5). **F** Western blotting assays of fibroblasts detected with anti-FN1, α-SMA, and FAP-1 antibodies after treatments with siNrf2 and exosomes. β-actin was used as an internal reference. Data were presented as the mean ± SEM from three independent experiments. *P < 0.05, **P < 0.01 compared with corresponding control; #*P* < 0.05, ##*P* < 0.01 compared with miR-1290 mimic or COX-2-exo groups

To identify the mechanims of exo-miR-1290 mediated CAFs activation, RNA sequence assays were employed to determine the biological process involved in the activation of CAFs mediated by exo-miR-1290. The results of Gene Ontology (GO) enrichment analysis showed that the antioxidant activities were involved in the COX-2 exosome-mediated CAFs activation (Fig. 4A, Additional file 3). It seemed that some antioxidant signal protein may play a major role in COX-2-mediated CAFs activation.

Nrf2 is a critical transcription factor that regulates the cellular antioxidant response by enhancing the expression of anti-oxidant genes [27]. Recently, it was reported that p62 Stimulated Nrf2 signaling during CAFs differentiation [28]. As Nrf2 is traditionally known for the role in redox homeostasis [27], we detected its expression during COX-2-mediated CAFs activation. Nrf2 protein was undetectable in NIH-3T3 cells. However, its level was elevated after treatment of A549-COX-2 exosomes (Fig. 4B). To clarify the role of Nrf2 on CAFs activation, Nrf2 was overexpressed in both MRC-5 and NIH-3T3 cells. Figure 4C shows that Nrf2 overexpression induced the upregulation of FAP-1, α-SMA, and FN1 expression. In contrast, siNrf2 suppressed miR-1290-mediated FAP-1 and FN1 expression. However, it had mild effects on the basal levels of the FAP-1 and FN1 protein, possibly due to the particularly low level of Nrf2 in fibroblasts (Fig. 4D, E). However, siNrf2 completely blocked CAFs activation and FN1 production mediated by A549-COX-2 exosomes (Fig. 4F). These data suggest that COX-2/exo-miR-1290 promotes CAFs activation by upregulating Nrf2 expression, and its upregulation is a crucial factor in exosomes-mediated CAFs activation and FN1 production in LUAD.

### Exo-miR-1290 upregulated CAFs activation by targeting CUL3 in fibroblasts

**Fig. 5:**
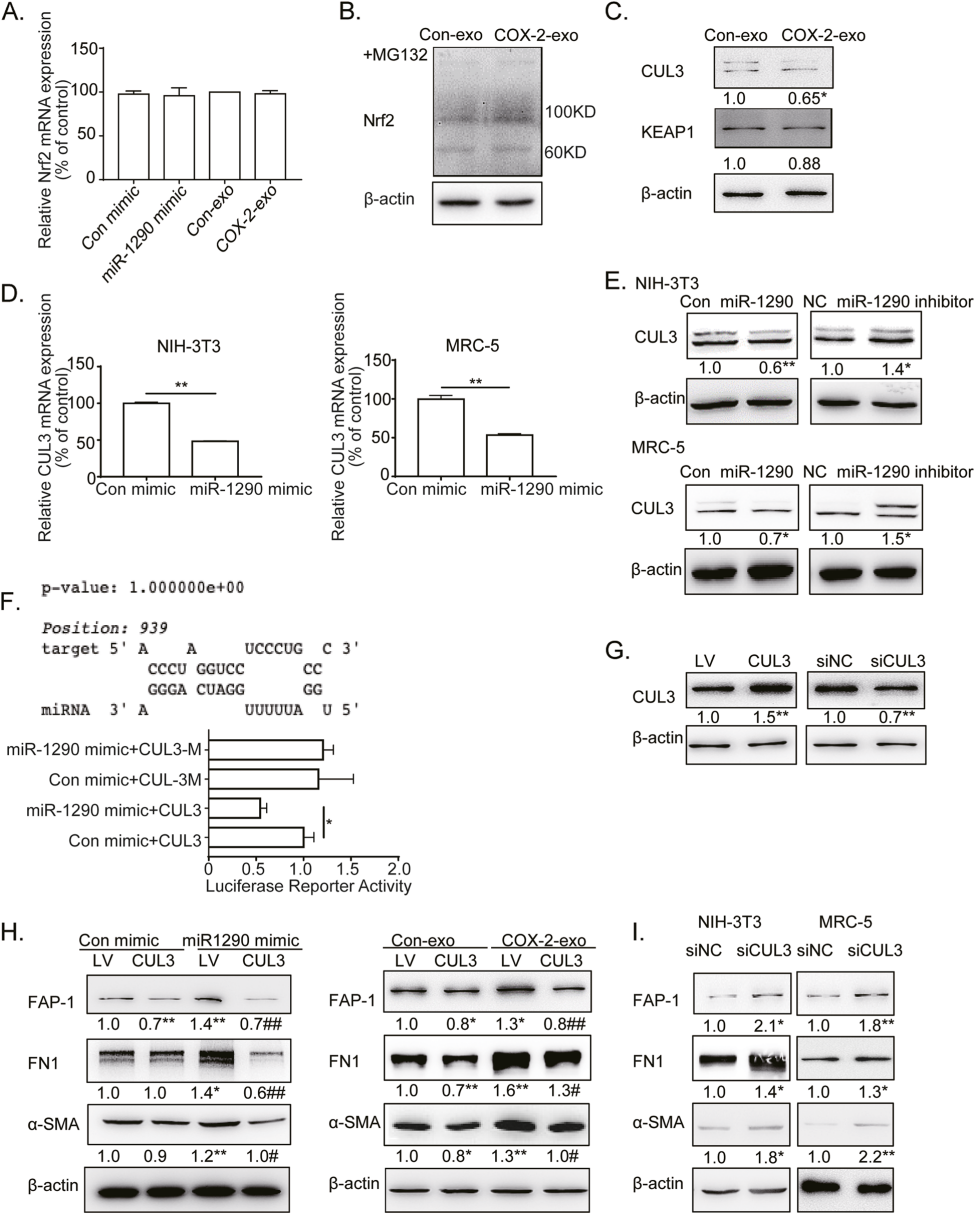
CUL3 was involved in exo-miR-1290-mediated Nrf2 upregulation and CAFs activation. **A** qRT-PCR assays of Nrf2 mRNA expression in NIH-3T3 cells treated with miR-1290 mimic or A549-COX-2 exosomes. **B** Western blotting assays of NIH-3T3 cells were detected with anti-Nrf2 antibody after treatments with MG132 and exosomes from A549-Con or A549-COX-2 cells. **C** Western blotting assays of NIH-3T3 cells detected with anti-CUL3 and KEAP1 antibodies after treatments with A549-con or A549-COX-2 exosomes. **E** Western blotting assays of fibroblasts detected with anti-CUL3 antibody after treatments with miR-1290 mimic or inhibitor. **F** The miR-1290 binding site of 3’UTR of CUL3 mRNA predicted by RNAhybrid 2.2 and miRWalk. Firefly luciferase reporter was used to analyze the activity of the miR-1290 binding site of the reporter with the wild-type CUL3 3’UTR (CUL3) or with the mutational CUL3 3’UTR (CUL3-M). **G** Western blotting assays of fibroblasts detected with anti-CUL3 antibody after CUL3 overexpression or siCUL3 treatments. **H** Western blotting assays of fibroblasts detected with anti- FN1, α-SMA, and FAP-1 antibodies after treatments with CUL3 overexpression and miR-1290 mimic transfection, or exosome. **I** Western blotting assays of fibroblasts were detected with anti- FN1, α-SMA, and FAP-1 antibodies after treatments with siCUL3. β-actin was used as an internal reference. Data were presented as the means ± SEM from three independent experiments. *P < 0.05, **P < 0.01 compared with corresponding control; #*P* < 0.05, ##*P* < 0.01 compared with miR-1290 mimic or COX-2-exo groups

To identify the mechanisms of Nrf2 upregulation in CAFs activation, the mRNA level of Nrf2 was detected by qRT-PCR. However, neither miR-1290 nor exosome treatment increased the Nrf2 mRNA level (Fig. 5A). These data suggest that COX-2/exo-miR-1290 may upregulate Nrf2 expression in a posttranscriptional pattern.

Subsequently, MG132 was used to inhibit the protease activities and prevent Nrf2 dagradation in fibroblasts. The data showed that MG132 canceled Nrf2 upregulation-mediated by A549-COX-2 exosomes (Fig. 5B), which let to the possibility that COX-2/exo-miR-1290 may upregulate Nrf2 expression by suppressing its degradation.

Nrf2 binds to KEAP1 and CUL3 to form an E3 ubiquitin ligase complex, which subjects Nrf2 to rapid proteasomal degradation in the cytoplasm [29]. Therefore, the expressions of KEAP1 and CUL3 were detected after treatment with exosomes derived from A549-Con or A549-COX-2 by western blotting. The results showed that CUL3, rather than KEAP1, was observed to be downregulated in this process (Fig. 5C). miR-1290 mimic downregulated both CUL3 mRNA and protein level in MRC-5 and NIH-3T3 cells, while miR-1290 inhibitor upregulated CUL3 expression (Fig. 5D, E). Consequently, these data prove that CUL3 is involved in exo-miR-1290-mediated Nrf2 regulation.

By bioinformatic assays, we found CUL3 was one of potential target of miR-1290. To confirm this, luciferase reporter constructs were made, which contain the putative binding site of the CUL3 3’-UTR region or its nucleotide substitutions in the 3’-UTR region (mutant). MiR-1290 overexpression inhibited the activity of the wild-type CUL3 reporter, while exhibiting no effect on the mutant region (Fig. 5F). These finding demonstrate that miR-1290 can specifically target the 3’-UTR regions of CUL3 by binding to its putative sequences.

To investigate the role of CUL3 in exo-miR-1290-mediated CAFs activation, NIH-3T3 cells were overexpressed with CUL3 by lentivirus infection to prepare 3T3-CUL3 stable cells. The CUL3 overexpression reversed miR-1290 mimic-mediated CAFs activation and FN1 expression. Similarly, CUL3 overexpression blocked the CAFs activation and FN1 expression induced by exosomes derived from A549-COX-2. On the contrary, CUL3 inhibition improved the CAFs activation and FN1 expression (Fig. 5G-I). These data suggest that CUL3 plays significant roles in COX-2/exo-miR-1290-mediated CAFs activation and FN1 production.

### Exo-miR-1290/CUL3 pathway upregulated CAFs activation by suppressing Nrf2 degradation and improving Nrf2/ARE transcriptional abilities in fibroblasts

**Fig. 6:**
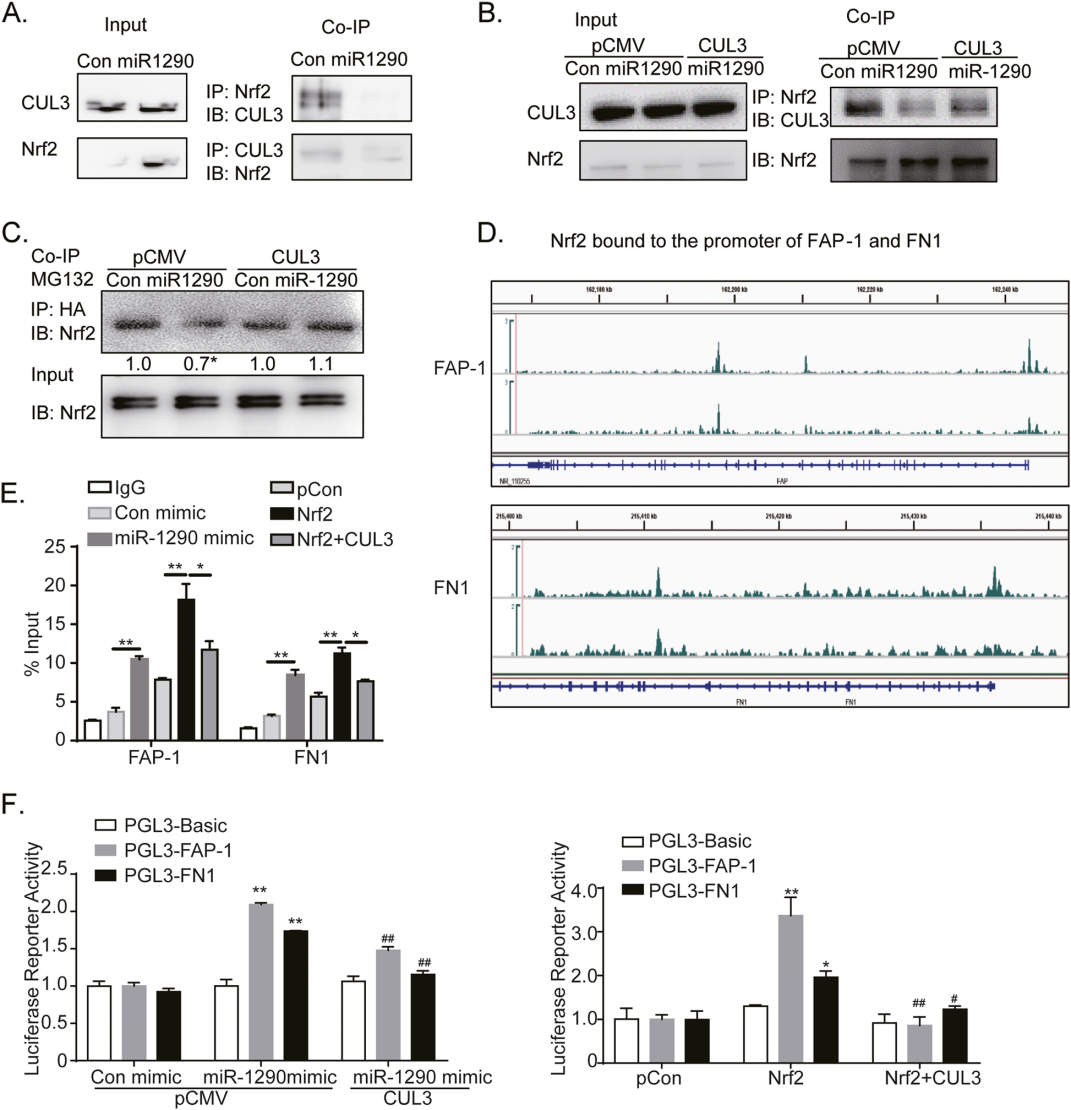
Exo-miR-1290 promoted CAFs activation by suppressing CUL3-mediated Nrf2 ubiquitination and enhancing Nrf2/ARE-mediated transcription activities. **A** Co-immunoprecipitation assays of NIH-3T3 cells detected with anti-Nrf2 and CUL3 antibodies after treatments with MG132 and miR-1290 mimic. **B** Co-immunoprecipitation assays of NIH-3T3 cells detected with anti-Nrf2 antibody after treatment with miR-1290 and CUL3 overexpression, followed by MG132 incubation. **C** Co-immunoprecipitation assays of Nrf2 ubiquitination in HEK-293 T cells detected with anti-HA antibody after transfection of miR-1290 mimic, CUL3-pCMV, and Ub-HA plasmids, followed by MG132 treatment. **D** Analysis of the promoters of FAP-1 and FN1 by JASPAR database. The promoter sequences from -2 K to + 1 K bp relative to our targeted TSS were scanned with FIMO (reference: https://meme-suite.org/meme/doc/cite.html) to search for NFE2L2 motif (MA0150.1) which was obtained from JASPAR database. To take advantage of different motif prediction methods, we also downloaded the predicted HOMER motif positions (http://homer.ucsd.edu/homer/index.html) and extracted positions for NFE2L (bZIP) and Nrf2 (bZIP) localized at targeted promoters. ChIP qPCR primers were designed to target regions with either NFE2L peaks or motifs. **E** ChIP assays of Nrf2 binding on the ARE domain in the promoters of FAP-1 and FN. HEK-293 T cells were treated with miR-1290 mimic, Nrf2, and CUL3 overexpression, then the cell lysis was incubated with Nrf2 antibodies, and qRT-PCR was used to confirm the binding of Nrf2 on the promoters of FAP-1 and FN1. **F** Luciferase reporter assays of transcriptional activities of FAP-1 and FN1. The Nrf2 binding site (ARE domain) of the promoters of FAP-1 and FN1 were cloned in PGL3-basic plasmids. Firefly luciferase reporter assays were used to analyze the transcriptional activities of FAP-1 and FN1 in HEK-293 T, after treatment with miR-1290 mimic, Nrf2, and CUL3 overexpression. Data were presented as the means ± SEM from three independent experiments, *P < 0.05, **P < 0.01, compared with corresponding control groups; #*P* < 0.05, ##*P* < 0.01, compared with miR-1290 mimic or Nrf2 overexpression groups

To determine whether Nrf2 stability is modulated by exo-miR-1290 pathway, the co-immunoprecipitation assays were used to detect the binding of CUL3 and Nrf2 in fibroblast cells after the treatment with miR-1290. The miR-1290 mimic suppressed the binding of CUL3 and Nrf2 complex, and CUL3 overexpression partially reversed miR-1290-suppressed binding (Fig. 6A, B). Considering the ubiquitin (Ub)-editing degradation of Nrf2, we examined the ubiquitylation pattern of Nrf2 in HEK-293 T cells. The Ub-HA plasmid was co-transfected with miR-1290 mimic or CUL3-pCMV plasmid. The results of co-immunoprecipitation experiments revealed that miR-1290 induced weaker bands, which corresponding to Nrf2 ubiquitination; whereas CUL3 overexpression reversed miR-1290-reduced Nrf2 ubiquitination (Fig. 6C). Therefore, these data suggest that miR-1290 suppresses CUL3 binding to Nrf2 and prevents Nrf2 degradation by ubiquitination.

Several studies have shown that Nrf2 triggers the transcription of multiple oncogenes by binding to the ARE domain of the promoters of these genes [29, 30]. In light of this, we conducted bioinformatic assays to identify the promoters of FAP-1, α-SMA and FN1. The bigwig files for NFE2L ChIP-seq were obtained from ENCODE project. There are several ARE domains in the promoters of FAP-1 and FN1 (Fig. 6D). The ChIP assays confirmed the presence of Nrf2 binding sites in the FAP-1 and FN1 promoter regions. Figure 6E shows that both miR-1290 mimic and Nrf2 transfection promoted the Nrf2 binding on the ARE domains of FAP-1 and FN1 promoters. However, CUL3 overexpression reversed the direct binding caused by Nrf2 overexpression.

To confirm whether miR-1290 or Nrf2 was responsible for FAP-1 or FN1 transcription, we constructed luciferase reporter plasmids encoding FAP-1 or FN1 promoters, respectively. The plasmids were transfected into HEK-293 T cells. Moreover, luciferase reporter assays showed that miR-1290 mimic and Nrf2 overexpression increased the transcriptional activities of FAP-1 and FN1, while CUL3 overexpression significantly reduced miR-1290 or Nrf2-mediated transcriptional activities (Fig. 6F).

Collectively, these data demonstrate that exo-miR-1290 elevates Nrf2-mediated transcription of FAP-1 and FN1 by suppressing CUL3 expression and CUL3-mediated Nrf2 degradation, eventually contributing to CAFs activation.

### COX-2/exo-miR-1290 promoted CAFs activation and tumor progression in vivo

In order to investigate the effects of COX-2/exo-miR-1290/CUL3 pathway on CAFs activation and tumor progression in vivo, we established tumor xenografts in nude mice by subcutaneously implanting A549-COX-2 cells and NIH-3T3 cells with or without stably expressing CUL3. The mice were subsequently euthanized 24 days after the cell injection, and tumors were resected.

**Fig. 7:**
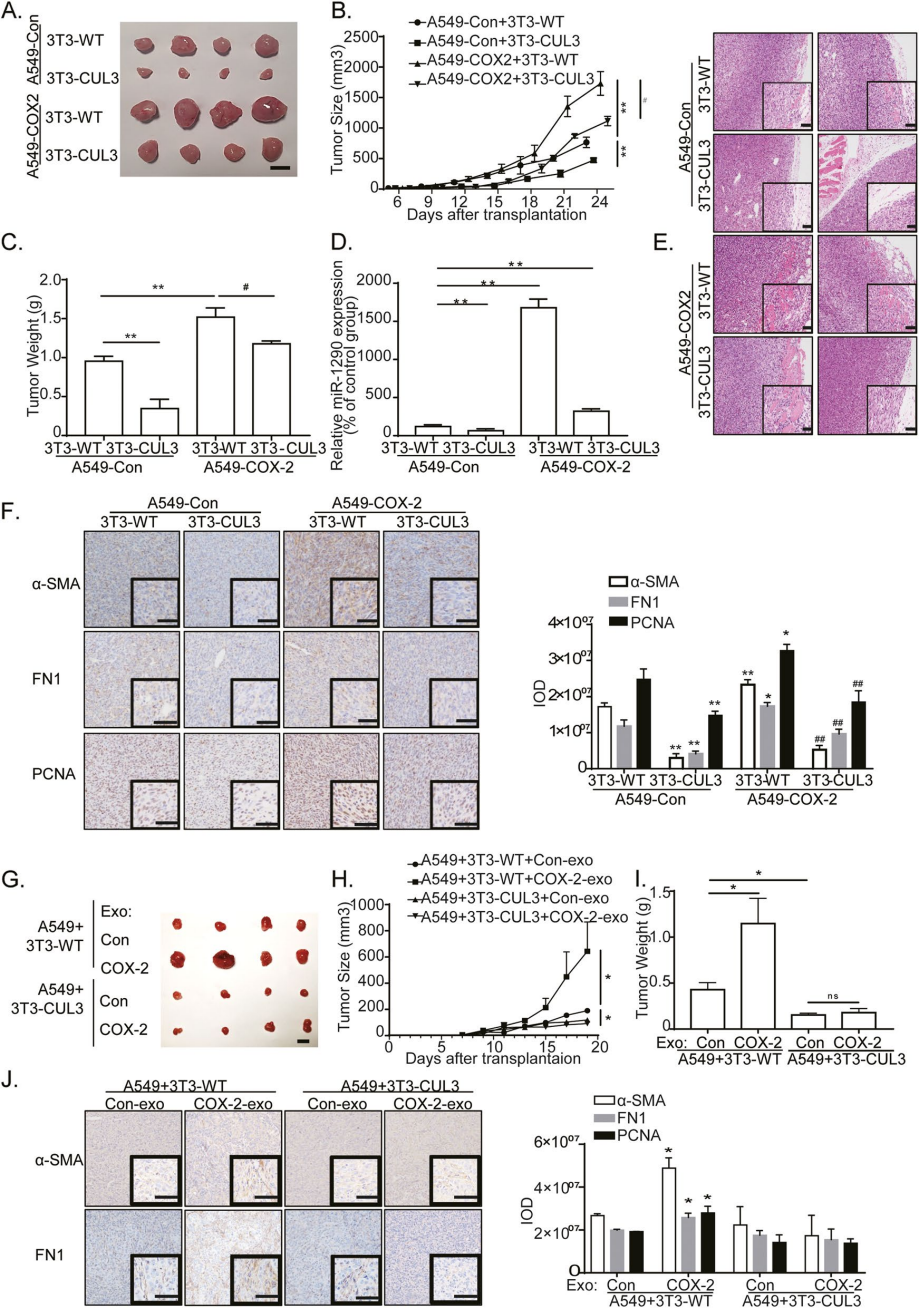
COX-2/Exo-miR-1290 promoted tumor progression and CAFs activation by CUL3/Nrf2 pathway in vivo. **A**-**F** CUL3 overexpression in fibroblasts reversed COX-2-mediated CAFs activation and tumor progression in vivo. Representative pictures of tumors (**A**), tumor size (**B**), and weight (**C**) were obtained at the indicated time point or following 24 days after the initial tumor cell injection (*n* = 8). Tumors were measured every 3 days and the tumor volumes were calculated. The tumors were excised and weighed after 24 days, and the representative pictures of tumors are displayed (Bar = 10 mm). Data are presented as the means ± SEM from all tumor samples. **D** qRT-PCR assays of exo-miR-1290 in plasma in each group. **E** HE staining of tumor tissues. Cancer cells invaded adjacent tissue mildly in the A549-Con + 3T3-WT group. There was no invasion in the A549-Con + 3T3-CUL3 group. However, Cancer cells invaded adjacent tissue significantly in the A549-COX-2 + 3T3-WT group, and the invasion was partially suppressed in the A549-COX-2 + 3T3-CUL3 group, compared with the A549-COX-2 + 3T3-WT group. **F** Immunohistochemistry staining of tumor tissues with antibodies of FN1, α-SMA, and PCNA (Low power view 10 × , High power view 40 × , Scale bars = 50 μm). **G**-**J** CUL3 overexpression in fibroblasts reversed exosome-mediated CAFs activation and tumor progression in vivo. Representative pictures of tumors (**G**), tumor size (**H**), and weight (**I**) were obtained at the indicated time points (*n* = 8). Various exosomes were given on day 11, 13, 15, 17 I.T. The tumors were excised and weighed after 20 days, and the representative pictures of tumors are displayed (Bar = 10 mm). Data are presented as the means ± SEM from all tumor samples. **J** Immunohistochemistry staining of tumor tissues with antibodies of FN1, and α-SMA (Low power view 10 × , High power view 40 × , Scale bar = 50 μm). * P < 0.05, ** *P* < 0.01, compared with corresponding control groups; #*P* < 0.05, ##*P* < 0.01, compared with A549-COX2 + 3T3-WT groups

Analysis of implanted tumor xenografts revealed that the mice with A549-COX-2 and NIH-3T3-WT coinjection showed more obvious tumor growth and invasion. Furthermore, xenografts with NIH-3T3-CUL3 partially abrogated COX-2-mediated tumor growth and invasion. The findings of qRT-PCR assays showed that the exo-miR-1290 expression was increased greatly in A549-COX-2 injected tumors; Conversely, exo-miR-1290 expression was partially downregulated in A549-COX-2 + 3T3-CUL3 groups, which may attributed to the decreased tumor sizes in this group (Fig. 7A-E).

The results of immunohistochemistry assays proved that COX-2 overexpression significantly improved PCNA expression, which presents for cancer cells proliferation, and α-SMA and FN1 expression in mesenchyma. However, this upregulation was deleted following 3T3-CUL3 coinjection (Fig. 7F). Furthermore, COX-2 overexpression in parenchyma significantly reduced E-Cadherin expression, and promoted N-Cadherin and Vimentin expression; Meanwhile, these processes were partially suppressed by NIH-3T3-CUL3 coinjection (Additional file 4).

To elucidate the effect of exosomes on CAFs activation and tumor progression in vivo, the tumor xenografts were subcutaneously implanted with both A549 cells and NIH-3T3, with or without CUL3 overexpression, and followed by intratumor injection of A549-Con or A549-COX-2 exosomes. The exosomes from A549-COX-2 cells improved tumor growth and invasion, and α-SMA and FN1 expression in mesenchyma in tumor tissues coinjected with NIH-3T3-WT cells. However, it did not affect tumor tissues coinjected with NIH-3T3-CUL3 cells (Fig. 7G-J, Additional file 5). Additionally, E-Cadherin expression was reduced in implanted tumor masses after treatment with A549-COX-2 exosomes, when NIH-3T3-WT cells were co-injected. However, NIH-3T3-CUL3 coinjection completely abolished COX-2-exosomes-mediated E-cadherin downregulation. Similarly, NIH-3T3-CUL3 coinjection completely reversed COX-2-exosomes-mediated upregulation of N-Cadherin and Vimentin (Additional file 5). The loss of E-Cadherin and the upregulation of N-Cadherin and Vimentin have been identified as hallmarks of epithelial-to-mesenchymal transition (EMT). Our data reveal that CUL3 in fibroblasts plays a major role in EMT process induced by exosomes from A549-COX-2 cells.

Eventually, these outcomes demonstrate that COX-2/exo-miR-1290 pathway promotes CAFs activation and tumor progression by decreasing CUL3 expression in fibroblasts.

## Discussion

CAFs are the most common types of stroma cells that play crucial roles in cancer development and progression [6, 7, 24, 31]. Numerous studies have revealed that CAFs were correlated with tumor growth, chemoresistance and poor survival [22, 32]. These findings suggest that targeting CAFs could be a promising approach for the treatment of LUAD. They shape the TME in various cancer tissues by regulating ECM synthesis, ECM remodeling and angiogenesis, eventually improving tumor invasion, EMT process and metastasis [7, 33]. According to previous studies, fibroblasts in their normal state have been found to inhibit tumor growth. However, when they are stimulated by cancerous cells, they undergo a transformation into CAFs [23, 34]; and the presence of FAP-1 and α-SMA are commonly used as indicators of CAFs [35–37]. However, the precise mechanisms underlying CAFs activation are still unclear. Our studies highlight the potential roles of COX-2/exo-miR-1290-mediated signaling pathways as effective targets to regulate CAFs activation and tumor progression in LUAD.

COX-2 was highly expressed in various cancer cells and regarded as an important factor associated with carcinogenesis and progression [10, 11, 37]. COX-2 expression in mesenchyma was also reported to promote cancer development in some tumor masses [38]. However, in cases of LUAD, it was observed that COX-2 exhibited a high level of expression in the parenchymal tissue, as opposed to the mesenchymal tissue [15, 39]. To this day, little is known about the effects of COX-2 in parenchyma on CAFs activation, let alone the underlying mechanism. The current data showed that higher expression of COX-2 in tumor cells was positively associated with CAFs activation. However, the related mechanism is still unknown. The present studies show that PGE2 has no effect on CAFs activation, suggesting that COX-2 may promote CAFs activation in a PGE2-independent manner.

Exosomes are essential components of the tumor microenvironment and act as messengers that transport signals between cells [17, 19]. Our data show that CAFs activation and ECM synthesis were improved by A549-COX-2 exosomes in vivo and vitro. It suggests that the exosomes play major roles in COX-2-mediated CAFs activation and ECM production in LUAD. Since exosomes contain a comprehensive contingent of functional proteins and miRNAs [21], we focus on identifying the specific components that undergo the CAFs activation mediated by COX-2, and the related mechanisms.

Several previous studies have demonstrated that exo-miRNAs improve the crosstalk between tumor cells and CAFs [7, 21]. To understand the specific exo-miRNAs involved in COX-2-mediated CAFs activation, we detected the expressions of 26 most common oncogenic miRNAs in lung cancer cells after treatment with COX-2 overexpression, and found that miR-1290 level was upreguated by COX-2, increasing about 100-fold in both cancer cells and exosomes. miR-1290 is well known as an oncogenic miRNAs in various cancers, including LUAD [25, 40–42]. MiR-1290 is increased in lung cancer cells and tumor tissues, which confer tumorigenicity, invasion and metastasis [25]. Additionally, serum exo-miR-1290 is significantly upregulated in LUAD patients compared to healthy controls, which is considered as an independent risk factor for the prognosis [26]. However, the impact of exo-miR-1290 on the activation of CAFs remains unclear.

In our studies, the basal level of miR-1290 was particularly low in fibroblast cells; while after the treatment with COX-2 exosomes, the expression level of miR-1290 in fibroblasts was significantly up-regulated. MiR-1290 mimic transfection induced CAFs activation and FN1 synthesis. On the contrary, miR-1290 ihhibtor suppressed these processes. Furthermore, the miR-1290 inhibitor blocked the CAFs activation and FN1 expression mediated by A549-COX-2 exosomes. These results identify that exo-miR-1290, which is induced by COX-2, plays a crucial role in CAFs activation and facilitates ECM production in LUAD. This discovery has unveiled a novel function of miR-1290 in tumor progression.

Using RNA sequence and bioinformatics assays, Nrf2, the antioxidant signal protein, was probably found to be a potential downstream of exo-miR-1290. The Nrf2 pathway is significantly activated in multiple cancers, including lung cancer [30, 43, 44]. Numerous evidences have established that the Nrf2 pathway in cancer cells drives cancer progression, metastasis, and chemoresistance [44–46]. The current study identified that Nrf2 expression was increased in CAFs after treatment with A549-COX-2 exosomes. Morever, Nrf2 exhibited similar effects on CAFs activation and ECM production as miR-1290, while the activation of CAFs mediated by exo-miR-1290 was reversed by siNrf2. These data suggest that the Nrf2 pathway is necessary for the COX-2/exo-miR-1290-mediated CAFs activation, thereby revealing a novel function of Nrf2 in stromal cells in the context of LUAD.

To further understand the mechanism of Nrf2 upregulation in exo-miR-1290-mediated CAFs activation, both the ubiquitination and the expression of Nrf2 were detected after treatment of miR-1290. Exo-miR-1290 improved Nrf2 expression by reducing its ubiquitination and degradation. In general, Nrf2 is degraded by the KEAP1-Nrf2-CUL3 E3 ubiquitin ligase complex [29, 30]. Using bioinformatic assays and luciferase reporter assays, CUL3 was found to be a potential target of miR-1290. CUL3 is an E3 ubiquitin ligase, an essential executor for regulating protein homeostasis in cancer development [47, 48]. However, the role and mechanisms of CUL3 in TME have not been reported. Herein, we hypothesized that exo-miR-1290 may suppress Nrf2 degradation in fibroblasts by targeting CUL3.

In our studies, miR-1290 mimic decreased CUL3 expression and then the interaction of CUL3 and Nrf2 complex, while CUL3 overexpression rescued miR-1290-reduced Nrf2 ubiquitination. Furthermore, CUL3 overexpression suppressed CAFs activation and ECM synthesis, and abrogated exo-miR-1290-mediated CAFs activation. Additionally, CUL3 overexpression in fibroblasts suppressed COX-2 exosomes-mediated cancer cell proliferation and invasion, in addition to ECM production and EMT process, and finally repressed tumor progression in vivo. These data demonstrate that CUL3 plays an essential role in prohibiting the transition of fibroblasts to CAFs in TME through degrading Nrf2, while COX-2/exo-miR-1290 pathway improves Nrf2 expression in CAFs through targeting CUL3 and preventing the CUL3-induced Nrf2 degradation (Additional file 6).

Therefore, we investigated the mechanisms of the CUL3-Nrf2 pathway that are implicated in COX-2/exo-miR-1290-mediated CAFs activation. The present research findings indicate that Nrf2 induced FAP-1 and FN1 transcription in CAFs by directly binding to their corresponding promoters. Furthermore, CUL3 overexpression blocked miR-1290-mediated FAP-1 and FN1 transcription through the degradation of Nrf2. Consequently, the CUL3-Nrf2 pathway may be a new target to overcome CAFs activation and ECM production, and eventually impeding tumor progression in LUAD.

## Conclusions

The objective of our study was to clarify that exo-miR-1290, which is induced by COX-2 overexpression, can promote CAFs activation and ECM production in LUAD. Consequently, we found that COX-2-mediated exo-miR-1290 improves CAFs activation and tumor progression by suppressing CUL3 expression and upregulating Nrf2 expression and Nrf2-mediated FAP-1 and FN1 transcription in fibroblasts. Our findings provide new insight into molecular mechanism of CAFs activation in LUAD progression. Targeting the COX-2/exo-miR-1290/CUL3/Nrf2 signal pathway might be a novel, stroma-focused, cancer prevention or treatment strategy.

### Supplementary Information

**Additional file 1.** The association of COX-2 expression and CAFs activation in LUSC.

**Additional file 2.** The effects of COX-2 inhibitor on the miR-1290 expression and CAFs activation.

**Additional file 3.** RNA sequence of NIH-3T3 after treatment of exosomes from A549-COX-2.

**Additional file 4.** The effects of COX-2 overexpression on EMT processes in A549+3T3 implanted tumor masses.

**Additional file 5.** The effects of exosomes from A549-COX-2 cells on invasion and EMT processes in A549+3T3 implanted tumor masses.

**Additional file 6.** The pathway drawn out as a schematic.

**Additional file 7.**

## Data Availability

The raw RNA-Seq data reported in this paper have been deposited in the Genome Sequence Archive (Genomics, Proteomics & Bioinformatics 2021) in National Genomics Data Center (Nucleic Acids Res 2022), China National Center for Bioinformation / Beijing Institute of Genomics, Chinese Academy of Sciences (GSA: CRA009594) that are publicly accessible at https://ngdc.cncb.ac.cn/gsa. Other datasets used and/or analysed during the current study are available from the corresponding author on reasonable request.

## Abbreviations

COX-2: Cyclooxygenase-2
CAFs: Cancer-associated fibroblasts
ECM: Extracellular mesenchyma
LUAD: Lung adenocarcinoma
LUSC: Lung squamous cell carcinoma
FAP-1: Fibroblasts activated protein-1
α-SMA: α-Smooth muscle actin
FN1: Fibronectin
PGE2: Prostaglandin E2
CUL3: Cullin 3
miRNAs: MicroRNAs
Nrf2: Nuclear Factor Erythroid 2–Related Factor 2
TME: Tumor microenvironments
FBS: Fetal bovine serum
PBS: Phosphate buffer solution
BCA: Bicinchoninic acid
SDS-PAGE: Sodium dodecyl sulfate–polyacrylamide gel electrophoresis
DiO: 3,3’-Dioctadecyloxacarbocyanine perchlorate
qRT-PCR: Real-time quantitative polymerase chain reaction
TSG101: Tumor susceptibility gene 101 protein
TCGA: The Cancer Genome Atlas
CD63: CD63 molecule
CD9: CD9 molecule
CD81: CD81 molecule
DMEM: Dulbecco’s modified Eagle’s medium
MEM: Minimum essential medium
Co-IP: Co-Immunoprecipitation
IOD: Integrated OD
ChIP: Chromatin immunoprecipitation assay
