# Supplementary figures and images for: Exo-miR-1290-induced by COX-2 overexpression promotes cancer-associated fibroblasts activation and tumor progression by CUL3-Nrf2 pathway in lung adenocarcinoma
Source: Cell Commun Signal. 2023 Sep 18;21:242. doi: 10.1186/s12964-023-01268-0 (PMC10506250; doi:10.1186/s12964-023-01268-0)

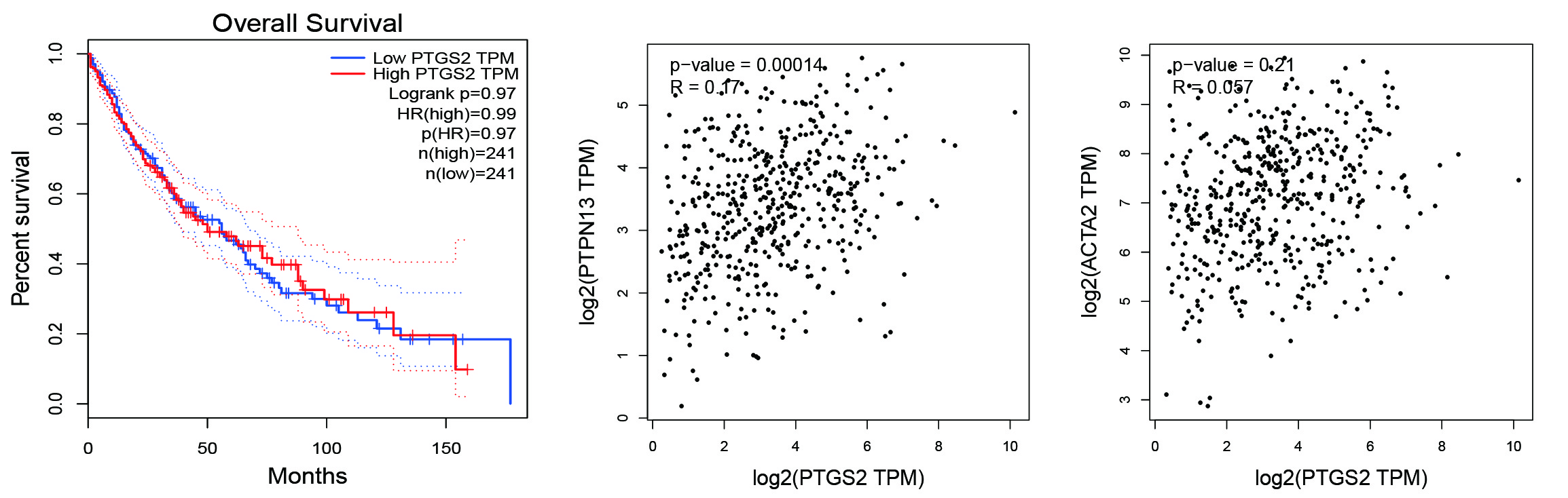

Supplement: Supplementary file 2 — Additional file 1. The association of COX-2 expression and CAFs activation in LUSC. [file 12964_2023_1268_MOESM1_ESM.tif]

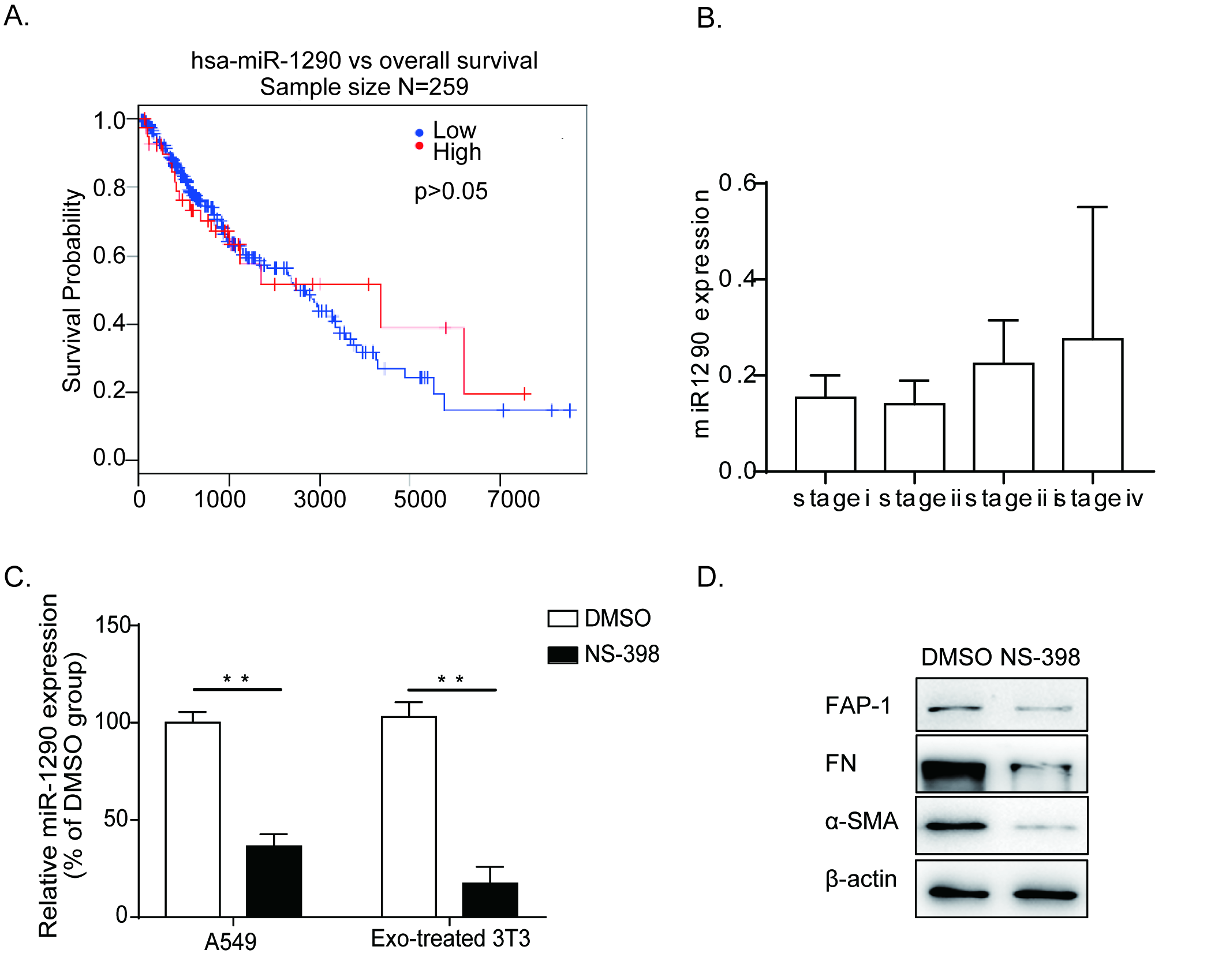

Supplement: Supplementary file 3 — Additional file 2. The effects of COX-2 inhibitor on the miR-1290 expression and CAFs activation. [file 12964_2023_1268_MOESM2_ESM.tif]

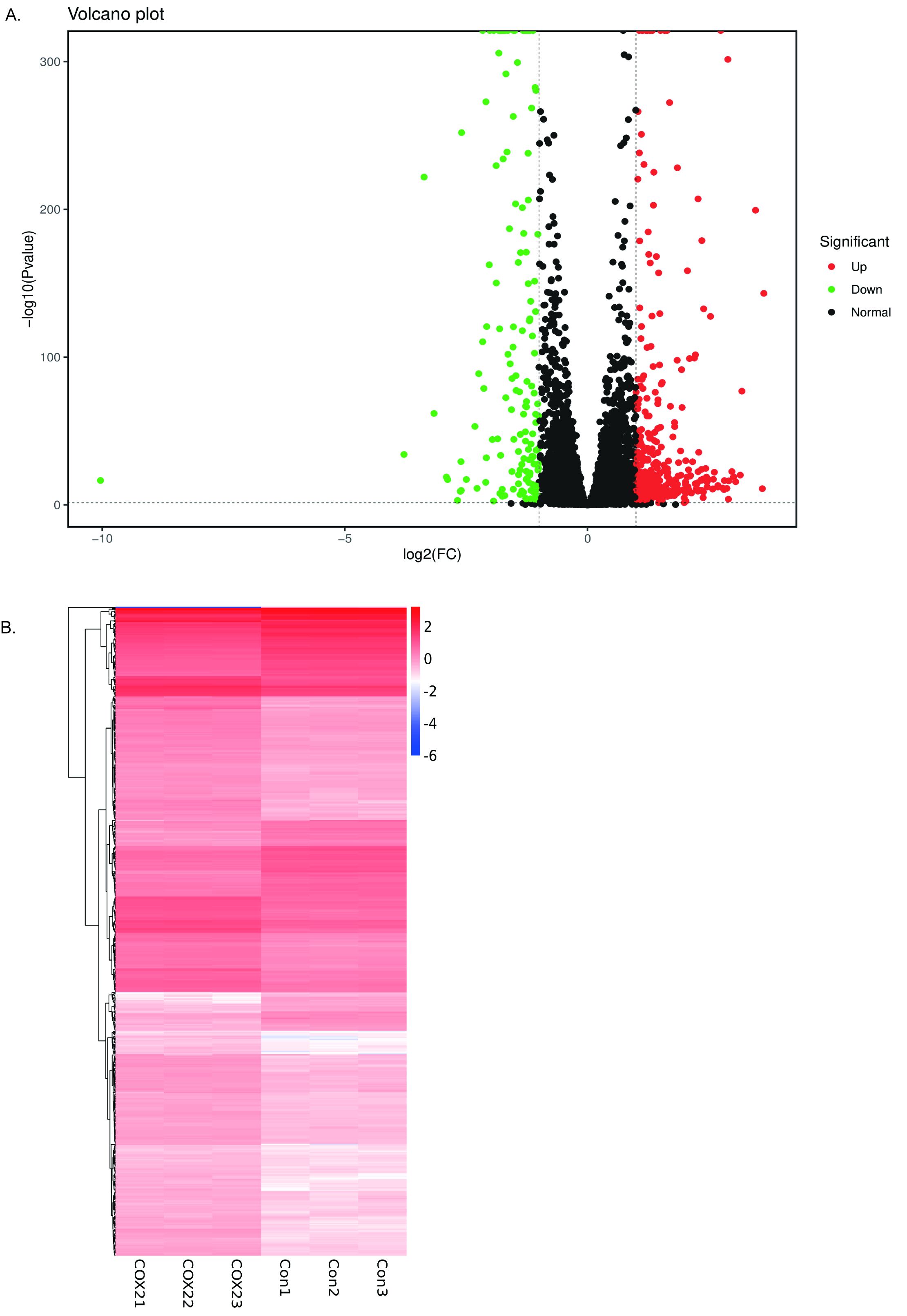

Supplement: Supplementary file 4 — Additional file 3. RNA sequence of NIH-3T3 after treatment of exosomes from A549-COX-2. [file 12964_2023_1268_MOESM3_ESM.tif]

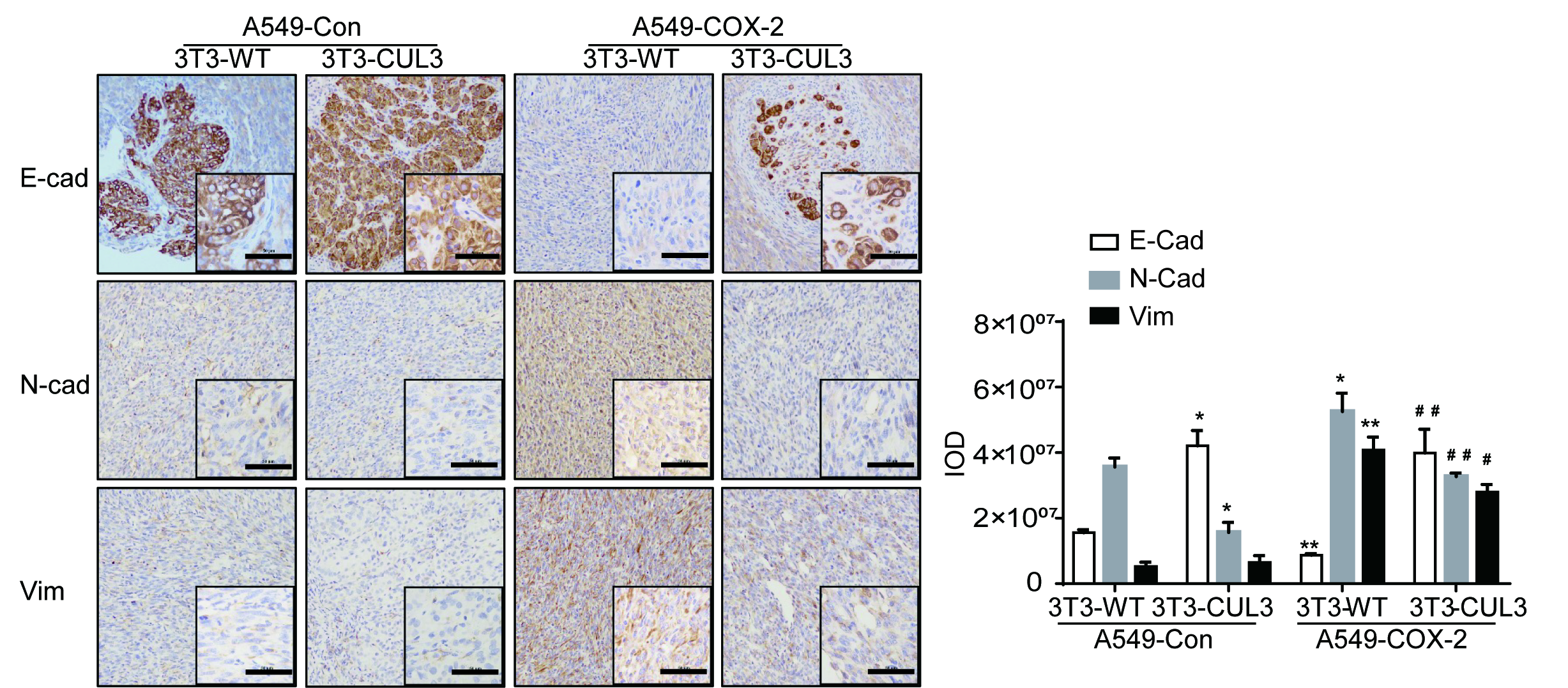

Supplement: Supplementary file 5 — Additional file 4. The effects of COX-2 overexpression on EMT processes in A549+3T3 implanted tumor masses. [file 12964_2023_1268_MOESM4_ESM.tif]

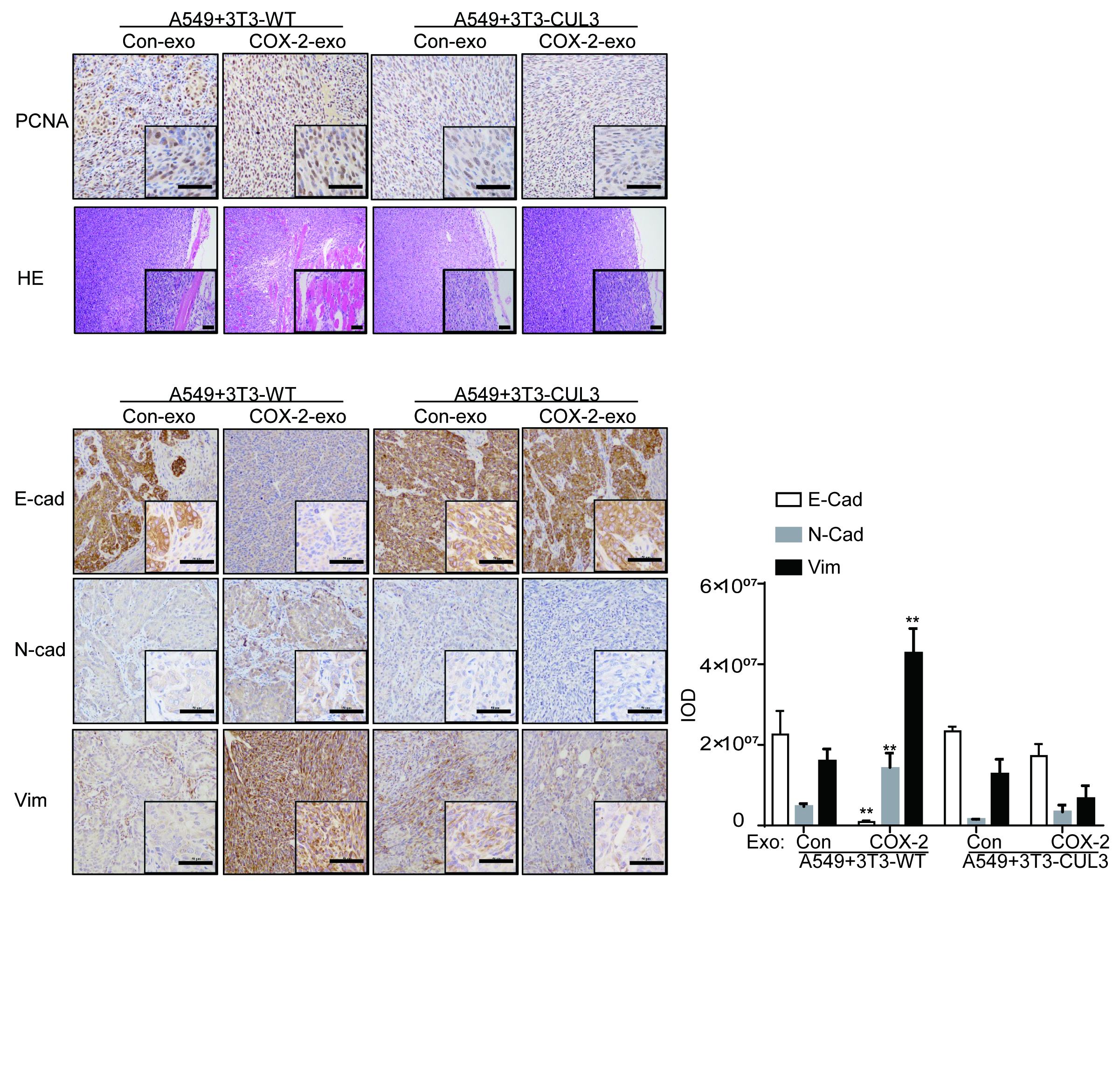

Supplement: Supplementary file 6 — Additional file 5. The effects of exosomes from A549-COX-2 cells on invasion and EMT processes in A549+3T3 implanted tumor masses. [file 12964_2023_1268_MOESM5_ESM.tif]

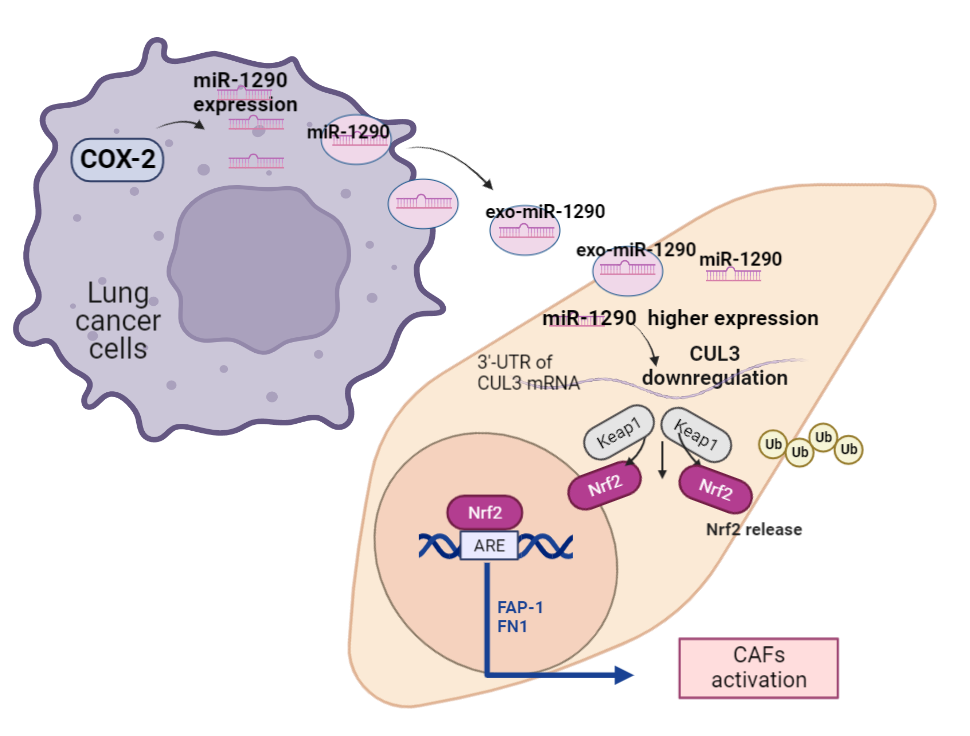

Supplement: Supplementary file 7 — Additional file 6. The pathway drawn out as a schematic. [file 12964_2023_1268_MOESM6_ESM.tif]
