# Supplementary material for: Exo-miR-1290-induced by COX-2 overexpression promotes cancer-associated fibroblasts activation and tumor progression by CUL3-Nrf2 pathway in lung adenocarcinoma
Source: Cell Commun Signal. 2023 Sep 18;21:242. doi: 10.1186/s12964-023-01268-0 (PMC10506250; doi:10.1186/s12964-023-01268-0)
Supplement: Supplementary file 8 — Additional file 7. [file 12964_2023_1268_MOESM7_ESM.docx]

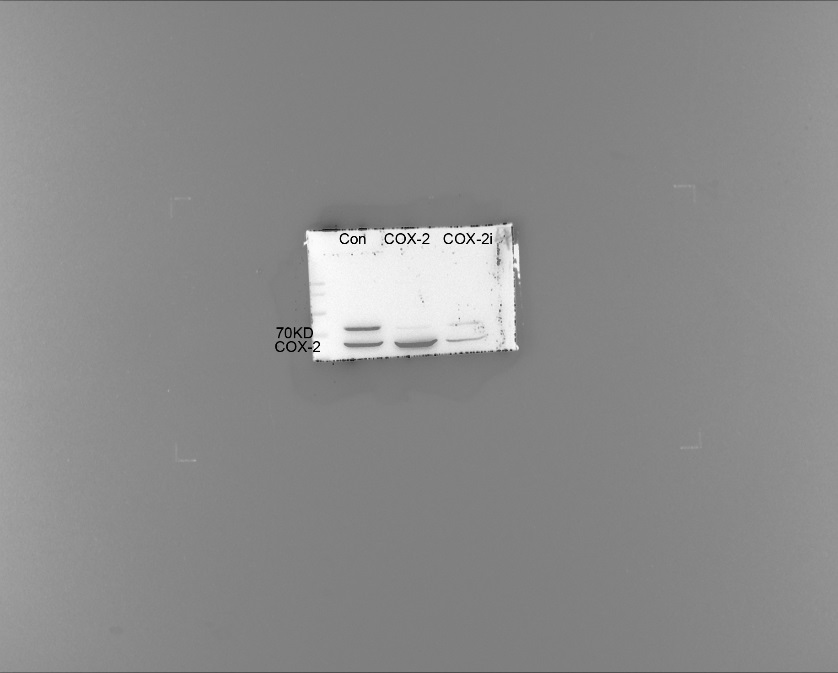

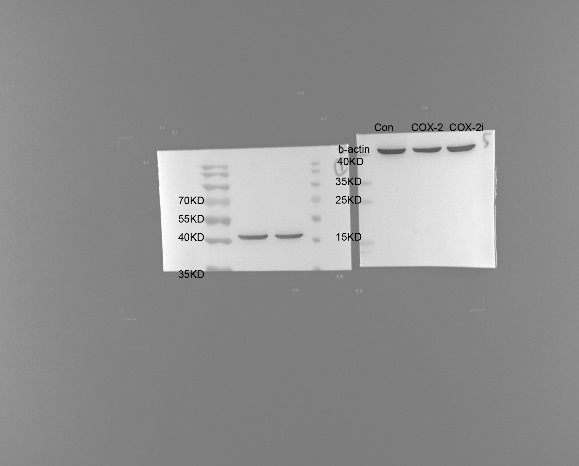


Fig. 1C. Overexpression of COX-2 in A549 cells were identified with Western bloting.


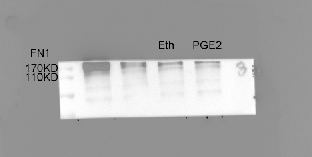

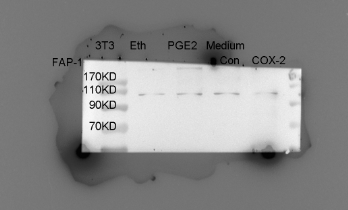


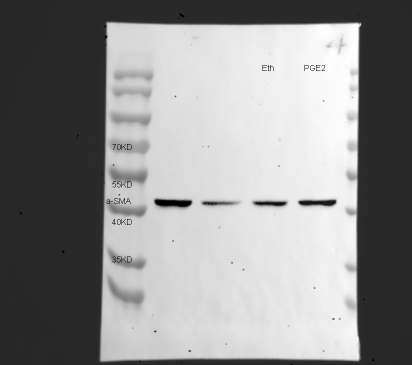

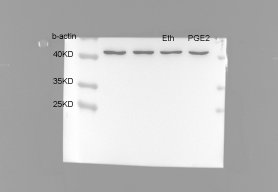


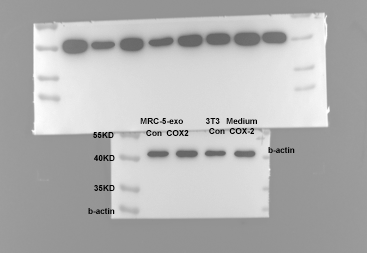


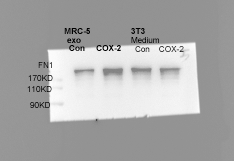

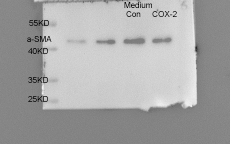


Fig.2 A Western bloting assays of NIH-3T3 cells detected with anti-α-SMA, FN1, and FAP-1 antibodies after treatments with PGE2 5 μM, or condition medium of A549-Con or A549-COX-2 cells for 48 h. β-actin was used as internal reference.


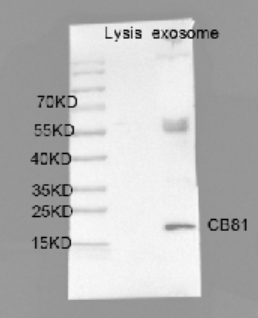

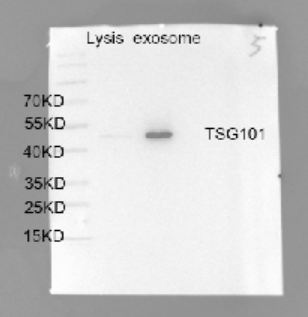

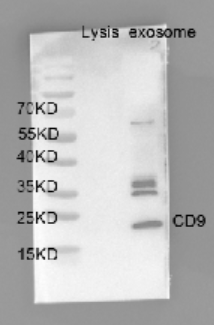


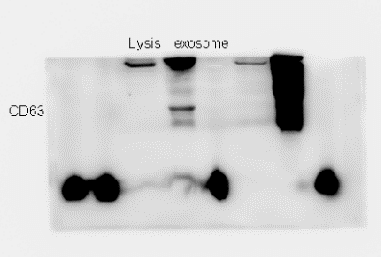


Fig. 2B. Exosome identification. Exosomes were identified by transmission electron microscope and the expressions of exosome markers (CD9, CD81, TGS101, CD63) were then assessed by Western bloting.


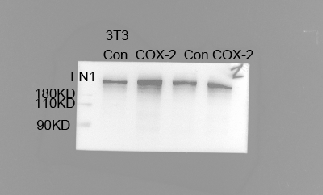

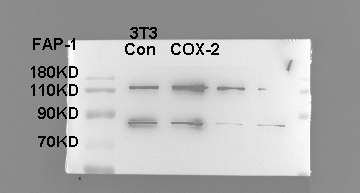


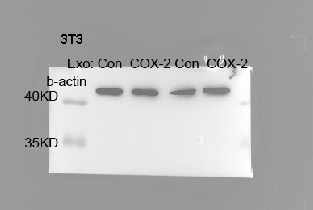

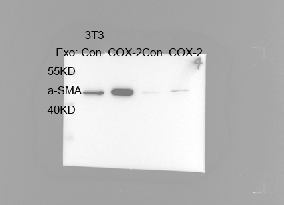


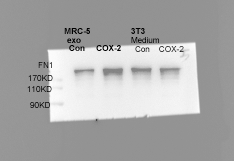

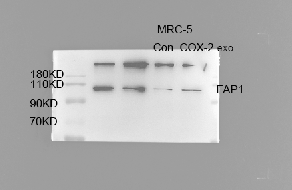

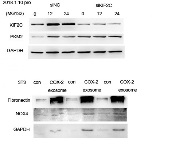




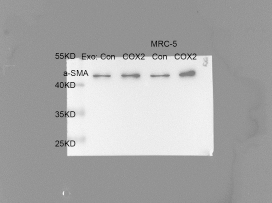

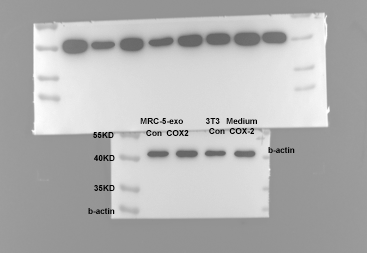


Fig. 2D. Western bloting assays of fibroblast cells detected with anti-α-SMA, FN, and FAP-1 antibodies after treatments with A549-Con or A549-COX-2 exosomes. β-actin was used as internal reference.


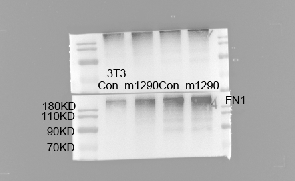

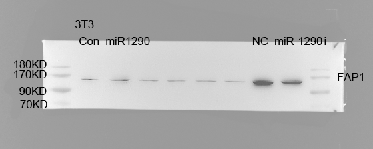


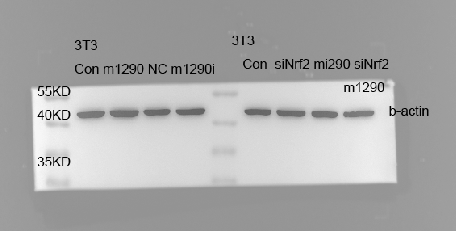

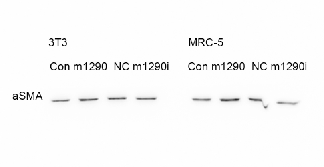


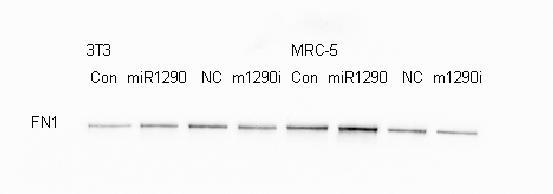

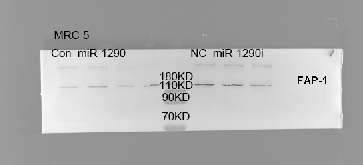


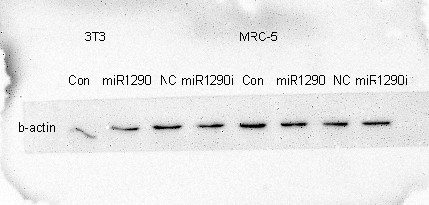


Fig. 3G. Western bloting assays of fibroblasts detected with anti-α-SMA, FN1, and FAP-1 antibodies after treatments with miR-1290 mimic or inhibitor, compared with corresponding control. β-actin was used as internal reference.


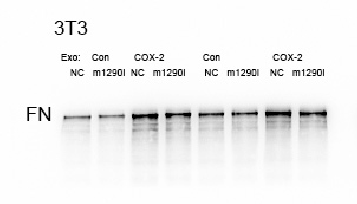

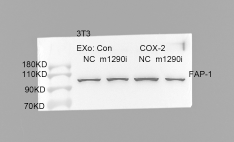


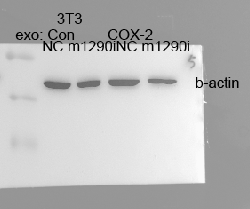


Fig. 3H. Western bloting assays of NIH-3T3 cells detected with anti-FN1 and FAP-1 antibodies after treatments with miR-1290 inhibitor and/or exosomes. β-actin was used as internal reference.


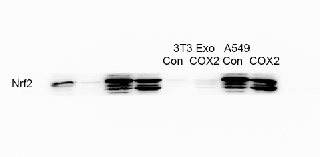


Fig. 4B. Western bloting assays of NIH-3T3 cells detected with anti-Nrf2 antibody after treatments with exosomes from A549-Con or A549-COX-2 cells.


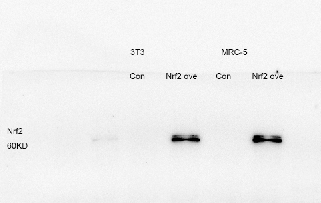

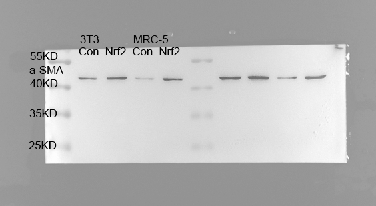


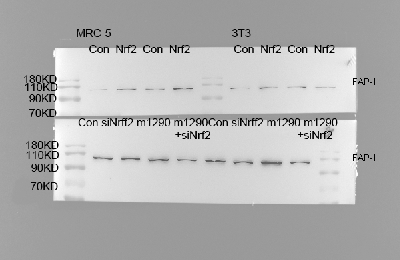

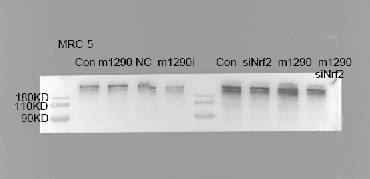


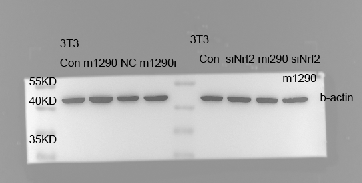

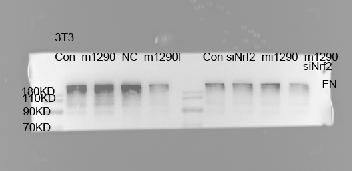


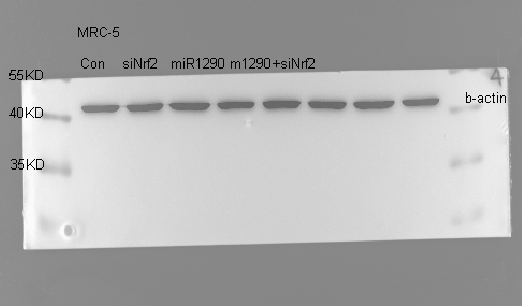


Fig. 4 C-E. Western bloting assays of fibroblasts detected with anti-α-SMA, FN1, and FAP-1 antibodies after treatments with Nrf2 overexpression. Western bloting assays of fibroblasts detected with anti-FN1, and FAP-1 antibodies after treatments with siNrf2 and/or miR-1290 mimic transfection.


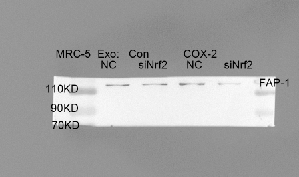


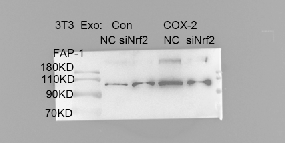


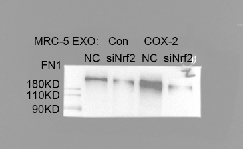

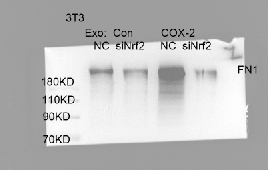


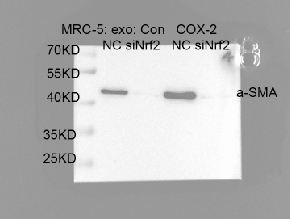

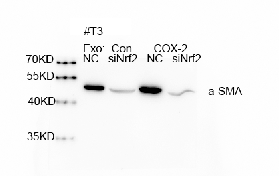


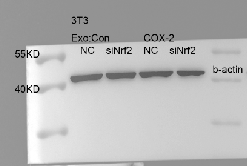

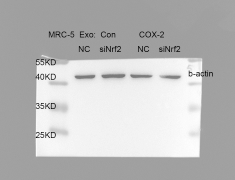


Fig. 4F. Western bloting assays of fibroblasts detected with anti-FN1, α-SMA, and FAP-1 antibodies after treatments with siNrf2 and exosomes. β-actin was used as internal reference.


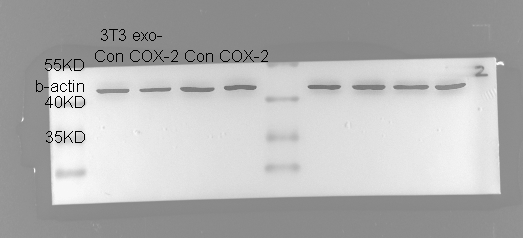

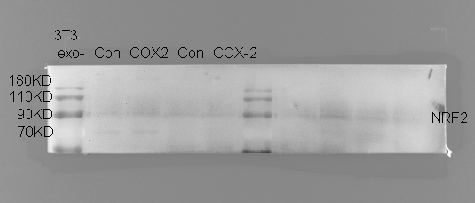


Fig. 5 B. Western bloting assays of NIH-3T3 cells detected with anti-Nrf2 antibody after treatments with MG132 and exosomes from A549-Con or A549-COX-2 cells. β-actin was used as internal reference.


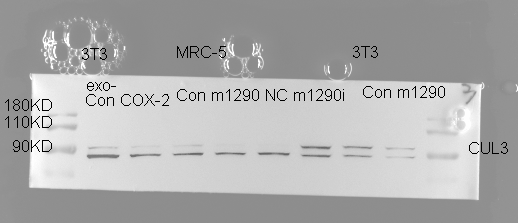

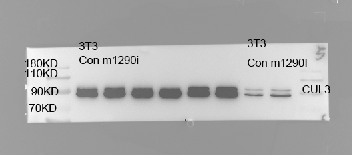


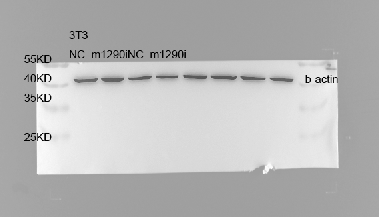

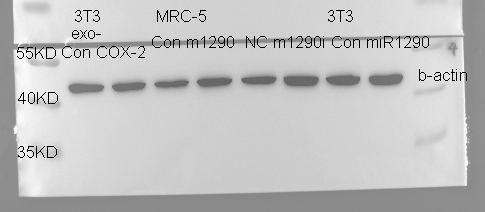


Fig. 5C. Western bloting assays of NIH-3T3 cells detected with anti-CUL3 antibodies after treatments with A549-con or A549-COX-2 exosomes. β-actin was used as internal reference.

Fig. 5E. Western bloting assays of fibroblasts detected with anti-CUL3 antibody after treatments with miR-1290 mimic or inhibitor. β-actin was used as internal reference.


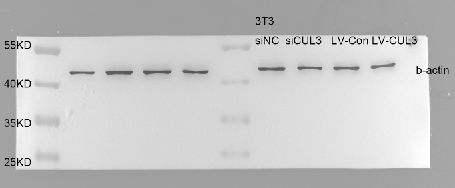

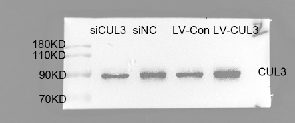


Fig. 5G. Western bloting assays of fibroblasts detected with anti-CUL3 antibody after treatments with CUL3 overexpression or siCUL3. β-actin was used as internal reference.


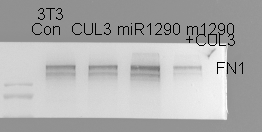

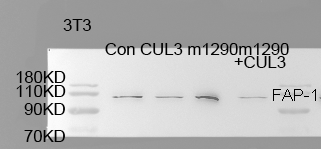


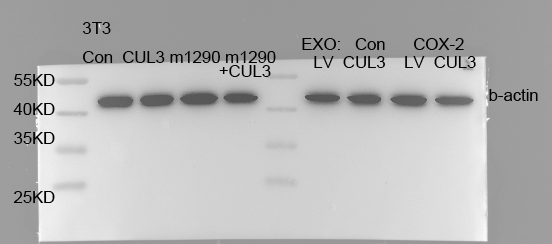

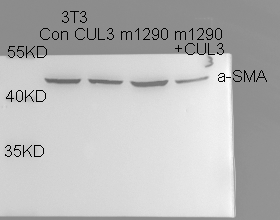


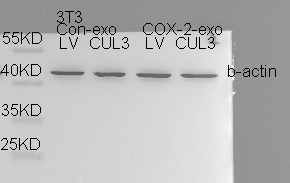

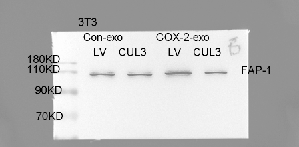


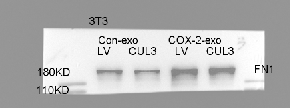


Fig. 5H. Western bloting assays of fibroblasts detected with anti- FN1, α-SMA, and FAP-1 antibodies after treatments with CUL3 overexpression and miR-1290 mimic transfection, or exosome. β-actin was used as internal reference.


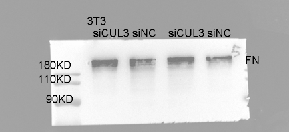


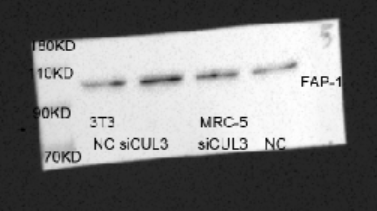


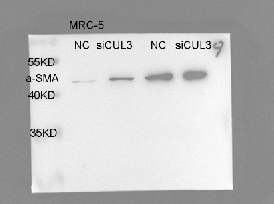

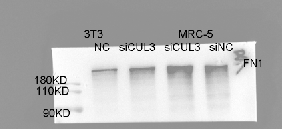


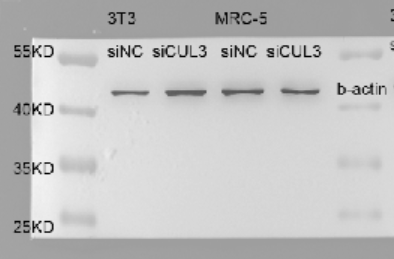

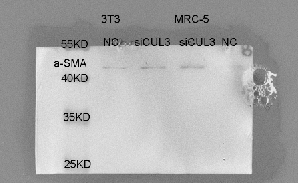


Fig. 5I. Western blotting assays of fibroblasts were detected with anti- FN1, α-SMA, and FAP-1 antibodies after treatments with siCUL3. β-actin was used as an internal reference.


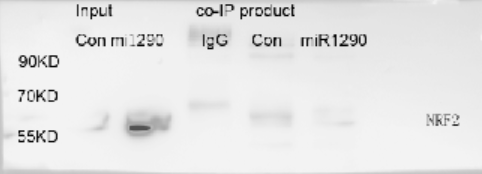

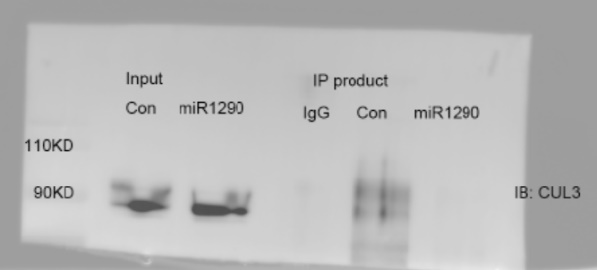


Fig. 6A. Co-immunoprecipitation assays of NIH-3T3 cells detected with anti-Nrf2 and CUL3 antibodies after treatments with MG132 and miR-1290 mimic.


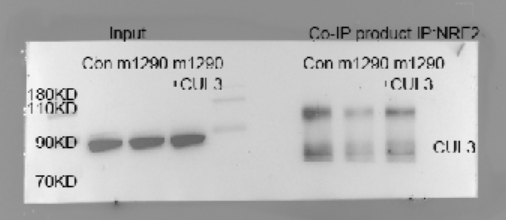


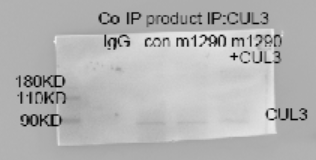

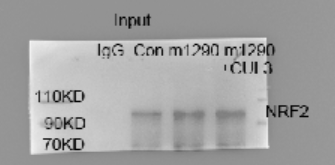


Fig. 6B. Co-immunoprecipitation assays of NIH-3T3 cells detected with anti-Nrf2 antibody after treatment with miR-1290 and CUL3 overexpression, followed by MG132 incubation.


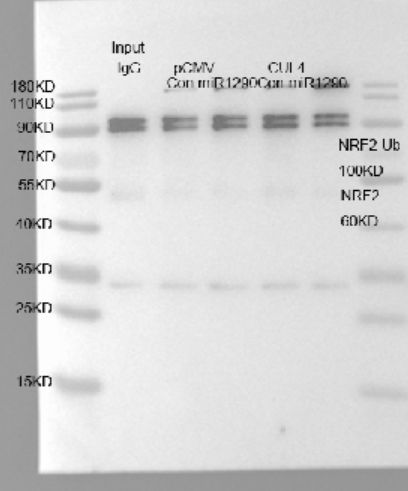

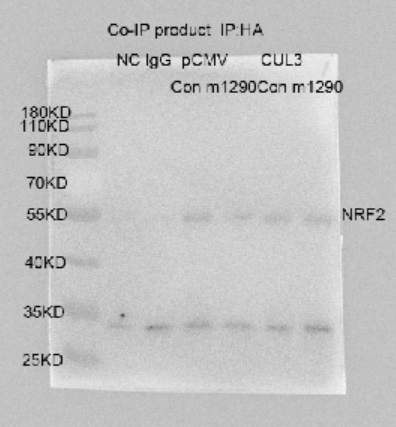


Fig. 6 C. Co-immunoprecipitation assays of Nrf2 ubiquitination in HEK-293T cells detected with anti-HA antibody after transfection of miR-1290 mimic, CUL3-pCMV, and Ub-HA plasmids, followed by MG132 treatment.
